# Effects of arbuscular mycorrhizal fungus inoculation on the growth and nitrogen metabolism of *Catalpa bungei* C.A.Mey. under different nitrogen levels

**DOI:** 10.3389/fpls.2023.1138184

**Published:** 2023-02-23

**Authors:** Wei Chen, Xueli Mou, Panpan Meng, Juan Chen, Xiaan Tang, Guihua Meng, Kexu Xin, Yi Zhang, Chunyan Wang

**Affiliations:** College of Forestry, Northwest A&F University, Yangling, Shaanxi, China

**Keywords:** woody plant, *Rhizophagus intraradices*, growth characteristics, physiological performance, nitrogen nutrition, gene expression

## Abstract

Evidence suggests that arbuscular mycorrhizal fungi (AMF) may promote the growth of woody plants. However, the effects of AMF on nitrogen (N) metabolism in plants, especially trees, and its regulatory mechanism are rarely reported. Here, the effects of AMF inoculation on the growth and N nutrition status of *Catalpa bungei* under different N levels were reported. Three N levels (low, medium, high) and two mycorrhizal inoculation treatments (inoculation with *Rhizophagus intraradices* or not) were used with factorial design. The results showed that medium N could significantly improve the physiological metabolism and growth of *C*. *bungei* seedlings. However, when N was excessive, growth was significantly inhibited whether inoculated AMF or not. Compared with non-inoculated treatments, AMF inoculation could promote the absorption of N and P, improve photosynthesis under low to medium N levels, thus promoting the growth of seedlings. AMF changed the biomass allocation in seedlings by reducing the stem mass ratio and root/shoot ratio, and increasing the leaf mass ratio. At medium N levels, compared with non-inoculated treatment, AMF inoculation could significantly promote root growth by changing root hormone levels and improving root architecture and root activity. Under N addition, AMF inoculation could improve the absorption and assimilation of N by regulating the expression of key enzyme genes of N metabolism and nitrate transporter genes (*NRT2.4*, *NRT2.5*, *NRT2.7*) in roots, and enhancing the activities of the key enzyme of N metabolism. This study may provide a reference for the application of AMF in the cultivation and afforestation technology of *C. bungei* in Northwest China.

## 1 Introduction

Mycorrhizas are mutualistic symbionts formed by the roots of higher plants and a certain kind of fungi in the soil, among which arbuscular mycorrhizas (AM) are the most widely distributed in nature (Das et al., 2022). Arbuscular mycorrhizal fungi (AMF) are a kind of soil beneficial microorganisms (Govindarajulu et al., 2005; Saia and Jansa, 2022), widely distributed in the forest, grassland, farmland and other ecosystems (Zhao et al., 2014; Zhu et al., 2014). Under natural conditions, AMF can form mycorrhizal symbiosis with more than 80% of plants (Ma et al., 2022). After AMF symbiosis with plants, dense hyphae networks can be formed in rhizosphere soil and root cortex cells, which can expand the effective absorption range of the root system, accelerate the transport of mineral elements and water, promote the absorption of soil mineral elements by hosts, regulate metabolic activities in hosts, and promote plant growth (Yang et al., 2014; Li et al., 2022). In addition, this efficient symbiosis can also help host plants cope with a variety of stress, such as disease (Weng et al., 2022), drought (Yang et al., 2014; Chen et al., 2020), salt (Ma et al., 2022) and heavy metal pollution (Riaz et al., 2021).

Nitrogen (N) is an important component of chlorophyll, and the content of chlorophyll directly affects the photosynthetic products. In addition, N is also an indispensable component of proteins, nucleic acids, enzymes and phytohormones in plants (Evangelina et al., 2015; Wang et al., 2021a). In particular, plant hormones, as important signaling substances, play a key role in plant growth, development and physiological response, and often interact with each other in a dynamic equilibrium state in regulating a certain growth process of plants and the adaptation to adversity (Cui et al., 2020). N metabolism is an important physiological activity in plants, including N absorption, reduction and assimilation, amino acid metabolism and transport, which is regulated by the expression of a variety of genes (Huang et al., 2022; Ren et al., 2022). In plant cells, low- or high-affinity nitrate transporters (NRT) and ammonium transporter are mainly involved in the direct uptake of N (Duan et al., 2015). The main pathway of N assimilation is that ${\text{NO}}_{3}^{-}$ is firstly reduced to ${\text{NO}}_{2}^{-}$ under the action of nitrate reductase (NR), and then reduced to ${\text{NH}}_{4}^{+}$ by the action of nitrite reductase (NiR). ${\text{NH}}_{4}^{+}$ is assimilated by glutamine (Gln) and glutamate (Glu) through the glutamine synthetase/glutamate synthetase (GS/GOGAT) pathway, and then N-containing compounds such as protein and nucleic acid are synthesized under the action of transaminases (Qin et al., 2021; Huang et al., 2022). In addition, glutamate dehydrogenase (GDH) can synthesize Glu under the consumption of ${\text{NH}}_{4}^{+}$ and 2-oxoglutarate (Luo et al., 2013). Therefore, the activity of key enzymes in N metabolism directly reflects the strength of N metabolism in plants. Evidence suggests that AMF can transfer a large amount of N to host plant cells, improve the utilization efficiency of N in the soil, reduce the input of chemical fertilizer, and make a virtuous cycle of the soil ecosystem, thus playing an important role in plant N nutrition (Govindarajulu et al., 2005; Savolainen and Kytöviita, 2022). At present, this has been confirmed in vegetables (Roussis et al., 2022), crops (Tanaka and Yano, 2005) and forage grass (Kang et al., 2020; Khan et al., 2021), but the researches on trees are relatively few. Moreover, the effects of AMF on plant N metabolism and its regulatory mechanism are rarely reported.

*Catalpa bungei* C.A.Mey. is a valuable and high-quality timber species and a famous ornamental tree species in traditional cultivation in China (Lv et al., 2021; Jian et al., 2022). Because of its excellent wood quality and high economic value, *C*. *bungei* is widely used in furniture, architecture, technology and other industries (Zheng et al., 2017; Wang et al., 2022). It has strong adaptability and rapid growth, so it is not only conducive to vegetation restoration in Northwest China to popularize and cultivate in a large area, but also provides support for local economic construction (Chen et al., 2021). In recent years, the researches on the fertilization of *C*. *bungei* have made some progress, mainly by increasing the application of N fertilizer to improve the growth of *C*. *bungei* plantation (Wu et al., 2015; Wang et al., 2021a). However, it not only requires higher economic costs, but also causes serious environmental pollution (Wu et al., 2017a; Sun et al., 2021). Therefore, it is an important factor to restrict the growth of *C*. *bungei* that how to apply fertilizer rationally to reduce the waste of resources and improve the efficient and rational use of N fertilizer. In addition, our previous research also showed that under drought stress, AMF could form a symbiotic relationship with *C*. *bungei*, improve water and nutrient status, and thus improve the drought resistance of *C*. *bungei* (Chen et al., 2020), which further confirmed the potential of AMF as microbial fertilizer. However, the inoculation effect of AMF may be related to a variety of factors, such as plant species, the genus or species of AMF colonizing roots, and N availability (Wu et al., 2017b), the effects of AMF inoculation on the growth of *C*. *bungei* under different N application levels have not been reported. Understanding the effects of AMF inoculation on woody plants under different nutrient levels is of great significance for further exploring the effects of the external environment regulating mycorrhizal symbiosis on tree phenotypic traits. Therefore, in this study, the effects of AMF inoculation on the growth, photosynthesis, nutritional status, N metabolism and expression of key genes of *C*. *bungei* were compared under different N concentrations under potted greenhouse conditions, to explore the effects of N application levels on AMF inoculation. The results can provide a theoretical basis for the development of AMF as microbial fertilizer, and also provide a reference for the application of AMF in the cultivation and afforestation technology of *C*. *bungei* in Northwest China.

## 2 Materials and methods

### 2.1 Mycorrhizal inoculum, test plant and cultivation substrate

The AM fungus *Rhizophagus intraradices* (BGC BJ09) was provided by the Institute of Plant Nutrition and Resources, Beijing Academy of Agriculture and Forestry Sciences, China. Before inoculation, maize was used as the host plant for propagation, and the inoculum consisted of a sand soil mixture containing spores, mycelium and infected root segments. Plant material, namely tissue culture seedlings of *C*. *bungei* “Jinsi” superior clone, was provided by the Henan Academy of Agricultural Sciences. The cultivation substrate was river sand. The screened river sand was washed with clean water (8–9 times), and naturally dried, then sterilized by high-pressure steam (121°C, 2 h) for reserve use.

### 2.2 Experimental design

The pot experiment was conducted in a two-factor completely randomized block design, including AMF inoculation and N application. Mycorrhizal inoculation included two levels: control treatment without inoculation (NAM) and inoculation with *R*. *intraradices* (AM). Three levels of N application were set (according to the screening results of the pre-experiment): 0.25 mM (low N), 10 mM (medium or moderate N) and 45 mM NH_4_NO_3_ (high N), respectively. A total of six treatments were formed, with 20 pots planted in each treatment and 0.75 kg of substrate in each pot, a total of 120 pots. The experiment was carried out in a glass greenhouse of Northwest A&F University.

The seedlings of *C*. *bungei* with the same growth trend were selected and transplanted into flowerpots (9 cm in diameter and 18 cm in depth, disinfected with 0.5% NaClO) with culture medium, and one plant was planted in each pot. In the inoculated treatment, 3 g of *R*. *intraradices* inoculum (approximately 200 spores per gram) was evenly spread on the roots of *C*. *bungei* seedlings during transplanting to fully contact with the roots. In the non-inoculated treatment, the same amount of inactivated inoculum (121°C, 2 h) was added in the same way, and 10 mL of inoculum filtrate (1 μm nylon net) was also added to ensure the consistency of microflora. After transplanting, the water supply was maintained normally (200 mL per plant per week), and Hoagland nutrient solution was supplemented every 10 days until the seedlings grew stably (about 45 days). The root samples of three seedlings were randomly selected to detect the colonization status of *R*. *intraradices*. After successful colonization, N application was started. 40 mL of 0.25 mM, 10 mM and 45 mM NH_4_NO_3_ solutions were irrigated respectively every 2 days for one month, during which Hoagland nutrient solution with N removed was supplemented every 10 days to ensure the normal supply of other nutrients. After fertilization treatment, the seedlings were normally supplied with water. After continuing to grow for one month, the seedlings were harvested and sampled for the determination of various physiological, biochemical and molecular biological indexes. The environmental conditions during seedling growth were as follows: day temperature, 20–35°C, night temperature, 10–20°C, a daylight cycle of 12 h, and relative humidity, 40%–85%.

### 2.3 Measurement of AMF colonization

The roots of seedlings were continuously washed with tap water, and then immersed in 10% KOH solution for 30 min in a water bath at 90°C. After the root segments were transparent, the residual KOH was rinsed with distilled water. Seedling roots were stained with Trypan-Blue (Phillips and Hayman, 1970), then observed with a 200× optical microscope (Olympus Bx43, Japan). The colonization rate of AMF was calculated according to the magnified intersections method (McGonigle et al., 1990).

### 2.4 Determination of gas exchange parameters and photosynthetic pigments

Before seedling harvest, Li-6400 portable photosynthetic apparatus (Li-COR, USA) was used to measure the gas exchange parameters of leaves, mainly including net photosynthetic rate (Pn, μmol·m^-2^s^-1^), stomatal conductance (Gs, mol·m^-2^s^-1^), transpiration rate (Tr, mmol·m^-2^s^-1^), and intercellular CO_2_ concentration (Ci, µmol·mol^-1^). The third, fourth and fifth leaves on the top of the seedlings were measured on a sunny morning (9:00–11:00). Instantaneous water use efficiency (WUE) was calculated by the ratio of Pn to Tr (Yang et al., 2014). 0.2 g of fresh leaves were weighed, chlorophyll (Chl a and Chl b) and carotenoids were extracted by the acetone direct extraction method, the absorbance at 663 nm, 646 nm, and 470 nm was measured and the pigment contents were calculated, respectively (Gao, 2006).

### 2.5 Determination of chlorophyll fluorescence parameters

The third, fourth and fifth leaves on the top of the *C*. *bungei* seedling were selected, and the chlorophyll fluorescence parameters were measured using a modulated chlorophyll fluorescence meter (MINI-Imaging-PAM, Walz, Germany) (Gao, 2006; Huang et al., 2018). The maximum photochemical efficiency (Fv/Fm), actual photochemical efficiency (ФPSII), potential photochemical efficiency of PSII (Fv/Fo), non-photochemical quenching (NPQ), photochemical quenching (qP) and relative electron transport rate (rETR) through PSII were calculated (Gao, 2006; Huang et al., 2018).

### 2.6 Growth parameters and biomass allocation

Leaves, stems and roots were harvested separately and then dried to a constant weight at 65°C after 15 min at 105°C, and the biomass (dry weight) was measured. The biomass allocation of each part was calculated according to the following formula (Xiao et al., 2015): leaf mass ratio (LMR) = leaf biomass/total biomass, stem mass ratio (SMR) = stem biomass/total biomass, root mass ratio (RMR) = root biomass/total biomass, root/shoot ratio = underground biomass/aboveground biomass.

### 2.7 Nutrient absorption and distribution

The dried root, stem and leaf samples were ground into fine powder by mortar. The N concentration in each part was determined by Kjeldahl, phosphorus (P) concentration was measured by the molybdenum-stibium colorimetric method, and potassium (K), calcium (Ca) and magnesium (Mg) concentrations were determined by flame photometric method, respectively (Bao, 2000).

### 2.8 Specific leaf area (SLA) and specific leaf weight (SLW)

A single leaf in the same position was collected and the single leaf area was measured using the transparent grid method (Gao, 2006). The leaf was then dried in the oven at 65°C to a constant weight and weighed. The SLA, SLW and leaf area per plant were calculated according to the following formula: SLA = leaf area (cm^2^)/leaf dry weight (g), SLW = leaf dry weight (g)/leaf area (m^2^), the leaf area per plant = total leaf dry weight (g)/SLW (g/cm^2^).

### 2.9 Root system architecture and root activity

Fresh seedling roots were cleaned under running water and then the root images were scanned with a digital scanner (Epson Expression 10000XL, Epson America, San Jose, CA, USA) at 300 dpi. Then the parameters such as root length, root surface area, average root diameter and root tips were calculated respectively by WinRHIZO root image analysis software (Version 2012b, Regent Instruments Inc., Montreal, QC, Canada) (Zheng et al., 2017). Root activity was determined by triphenyltetrazolium chloride (Gao, 2006).

### 2.10 Phytohormone levels

The contents of auxin (indole-3-acetic acid, IAA), gibberellin (GA_3_), cytokinin (CTK), and abscisic acid (ABA) in roots were determined by high performance liquid chromatography (Pan et al., 2010).

### 2.11 Determination of key enzyme activities in N metabolism

0.1 g of fresh root samples were fully ground in liquid N_2_. The activities of NR, NiR, GS, GOGAT and GDH in roots were measured using the corresponding enzyme-linked immunosorbent assay (ELISA) kits. The corresponding article numbers of the kits are JLC7018, JLC79203, JLC79209, JLC72932, and JLC72820, respectively. Before the enzyme activity assay, bicinchoninic acid (BCA) protein assay kits (JLC-SJ2508) were used to quantitatively measure the protein concentration of each sample. All the above kits were obtained from Shanghai Jingkang Bioengineering Co., Ltd. According to the instructions of the kit, sample addition, incubation (37°C, 30 min), washing, color development, and other processes were carried out. The absorbance was measured at 450 nm and the concentration of enzyme activity in the sample was calculated by the standard curve.

### 2.12 Expression analysis of genes related to N metabolism

100 mg of fresh root samples were snap-frozen in liquid N_2_ and ground into fine powder. The total RNA of the sample was extracted and purified by polysaccharide/polyphenol plant RNA Extraction Kit (NG3021S, HLingene; containing gDNA removal column). The concentration and quality of extracted RNA were determined using a micro nucleic acid protein analyzer (NanoDrop One, Fisher Scientific, USA). The first strand of cDNA was synthesized by reverse transcription kit (RT mix with DNase (All-in-One), US EVERBRIGHT INC), which was used as the template for RT-PCR. With reference to the preliminary transcriptome data (stored in NCBI/SRA database with the accession number PRJNA907402 (http://www.ncbi.nlm.nih.gov/bioproject/907402)) of *C*. *bungei*, some coding sequences (CDS) of genes related to N metabolism were screened, and gene-specific primers for qRT-PCR were designed using the online primer design tool Primer 3 (https://www.ncbi.nlm.nih.gov/tools/primer-blast/). The ubiquitin (UBQ) gene of *C*. *bungei* was used as the reference gene. The sequence information of specific primers was shown in **supplementary data (Table S1)**. The primers were synthesized by Sangon Biotech (Shanghai) Co., Ltd. qRT-PCR analysis was performed using CFX 96 real-time PCR instrument (Bio-Rad, Hercules, CA, USA) with differently treated cDNA as a template. Each treatment contained 3 biological replicates and 4 technical replicates. The relative expression levels of target genes were calculated by 2^-ΔΔCT^ method (Livak and Schmittgen, 2001).

### 2.13 Statistical analysis

SPSS 25.0 software was used for the statistical analysis of data. One-way analysis of variance (ANOVA) was used to analyze the different treatments, and then the Duncan test was used to make multiple comparisons. The significance level was set as α = 0.05. All data were expressed as mean ± standard error (SE). The effects of N application, inoculation and their interaction on the measured indexes were evaluated by two-way ANOVA. The Pearson correlation coefficient was used to evaluate the correlation between AMF colonization and other indicators. Using principal component analysis (PCA), the data were standardized and then computed by function rda () in the vegan library in R (http://www.r-project.org/). Sigmaplot 12.0 software was used for drawing.

## 3 Results

### 3.1 AMF colonization

**Figure 1:**
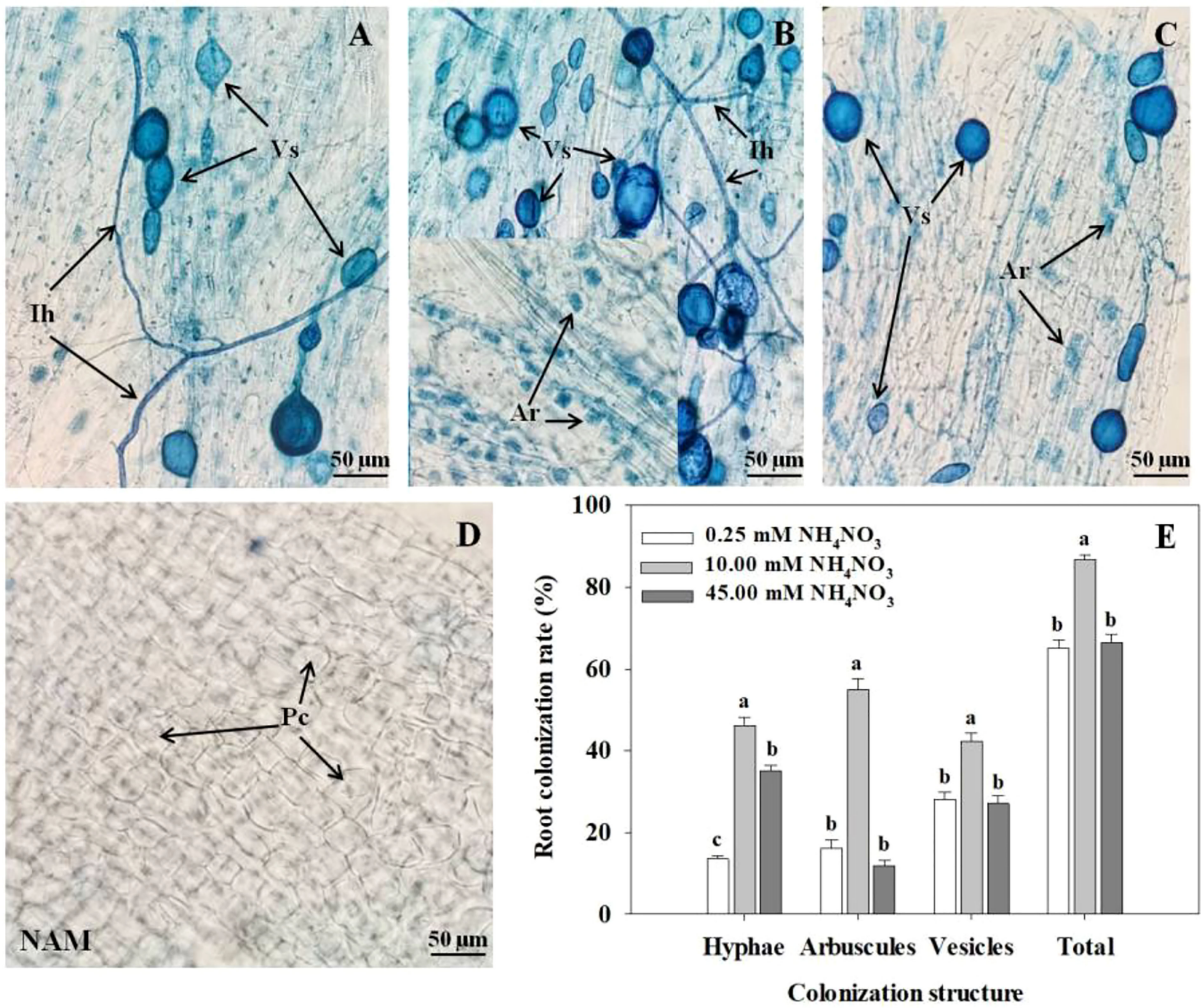
Colonization and development of arbuscular mycorrhizal fungus (AMF) *Rhizophagus intraradices* in *Catalpa bungei* seedling roots under **(A)** low N (0.25 mM), **(B)** moderate N (10 mM) and **(C)** high N (45 mM) levels. **(D)** The root of a non-inoculated plant. **(E)** Colonization rate of mycorrhizal *C. bungei* seedlings under different N levels. A, B, C and D, bars = 50 μm. Ih, intraradical hyphae; Vs, vesicles; Ar, arbuscules; Pc, plant cell; NAM, non-AMF-inoculated. Different lowercase letters above the bars indicate significant differences (*p* < 0.05) among different N levels. Values are means ± SE (*n* = 6).

After inoculation with *R*. *intraradices*, the colonization structures of *R*. *intraradices*, such as intraradical hyphae, arbuscules, and vesicles, can be clearly observed in roots at low N (**Figure 1A**), medium N (**Figure 1B**) and high N (**Figure 1C**) levels, while the colonization was not detected in each treatment without inoculation (**Figure 1D**). After AMF inoculation, with the increase of N concentration, the colonization rates of hyphae, arbuscules, vesicles and total colonization rate in roots of *C*. *bungei* seedlings increased significantly (*p* < 0.05), reached the maximum at medium N concentration, and decreased markedly at high N levels (**Figure 1E**).

### 3.2 Growth parameters

**Figure 2:**
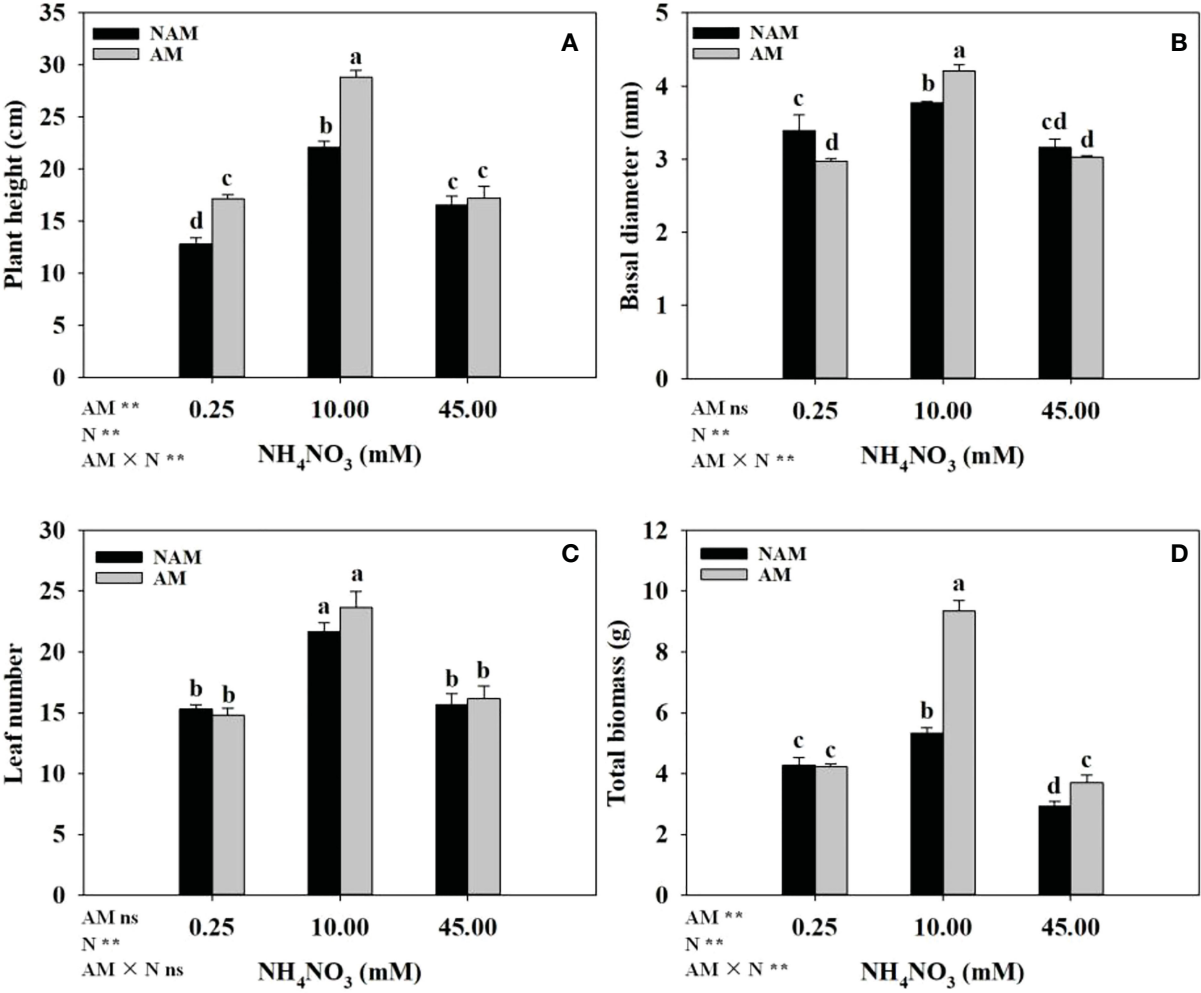
Effect of the arbuscular mycorrhizal fungus (AMF) *Rhizophagus intraradices* on **(A)** plant height, **(B)** basal diameter, **(C)** leaf number and **(D)** total biomass of *Catalpa bungei* seedlings under different nitrogen (N) levels. NAM, non-AMF-inoculated; AM, AMF-inoculated. Different lowercase letters above the bars indicate significant differences (*p* < 0.05) among treatments. Values are means ± SE (*n* = 6). Two-way ANOVA output: ns, not significant; *p < 0.05; **p < 0.01.

The growth of *C*. *bungei* seedlings was significantly affected by N application (**Figure 2**). Whether inoculated or not, with the increase of N concentration, the growth parameters (plant height, basal diameter, leaf number, total biomass) of *C*. *bungei* seedlings increased significantly, and reached the maximum at medium N levels, but decreased notably at high levels (**Figures 2A–D**). At low N levels, compared with non-inoculated treatment, AMF inoculation significantly increased the plant height and decreased the basal diameter of seedlings (*p* < 0.05), but had no significant effect on biomass accumulation. At medium N levels, the plant height (**Figure 2A**), basal diameter (**Figure 2B**), and total biomass (**Figure 2D**) of mycorrhizal seedlings were significantly higher than those of non-mycorrhizal seedlings (*p* < 0.05), which were 1.30-, 1.12- and 1.76-fold of those of non-inoculated seedlings, respectively. At high N levels, AMF inoculation notably increased the total biomass, but there were no significant differences in plant height and basal diameter between the inoculated and non-inoculated treatments. In addition, no significant difference in leaf number between inoculated and non-inoculated treatments was detected (**Figure 2C**).

### 3.3 Biomass allocation

The biomass allocation of *C*. *bungei* seedlings changed significantly after inoculation and N application (**Table 1**). Whether inoculated or not, the leaf biomass, stem biomass and SMR of *C*. *bungei* seedlings increased first and then decreased with the increase of N concentration, and the LMR increased gradually, while the RMR and root/shoot ratio decreased gradually. Among them, the leaf biomass of mycorrhizal or non-mycorrhizal seedlings reached a significant difference among the three N concentrations, and the stem biomass at medium N levels was significantly higher than that of low or high N levels (*p* < 0.05). Without inoculation, the root biomass was significantly decreased with the increase of N concentration, while it was firstly significantly increased and then markedly decreased after AMF inoculation. At the same N levels, the LMR > RMR > SMR, that is, the biomass of inoculated or non-inoculated seedlings tended to be allocated to leaves, followed by roots, and the proportion allocated to stems was the least (leaf > root > stem).

**Table 1:** Effect of the arbuscular mycorrhizal fungus (AMF) *Rhizophagus intraradices* on biomass allocation in different parts of the *Catalpa bungei* seedlings under different nitrogen (N) concentrations.

NH_4_NO_3_ (mM)	AMF status	Leaf biomass (g)	Stem biomass (g)	Root biomass (g)	Leaf mass ratio	Stem mass ratio	Root mass ratio	Root/shoot ratio
0.25	NAM	1.93 ± 0.12d	0.50 ± 0.03c	1.86 ± 0.18ab	0.45 ± 0.02e	0.12 ± 0.01b	0.43 ± 0.03a	0.78 ± 0.08a
	AM	2.13 ± 0.06d	0.44 ± 0.01cd	1.65 ± 0.12b	0.51 ± 0.02d	0.11 ± 0.00b	0.39 ± 0.02a	0.65 ± 0.06b
10.00	NAM	3.22 ± 0.14b	0.83 ± 0.03b	1.28 ± 0.03c	0.60 ± 0.01c	0.16 ± 0.01a	0.24 ± 0.01b	0.32 ± 0.01c
	AM	6.10 ± 0.21a	1.12 ± 0.03a	2.14 ± 0.12a	0.65 ± 0.00b	0.12 ± 0.00b	0.23 ± 0.01b	0.30 ± 0.01c
45.00	NAM	1.82 ± 0.12d	0.41 ± 0.02cd	0.71 ± 0.06d	0.62 ± 0.02bc	0.14 ± 0.00a	0.24 ± 0.02b	0.32 ± 0.03c
	AM	2.58 ± 0.18c	0.39 ± 0.04d	0.73 ± 0.06d	0.70 ± 0.01a	0.10 ± 0.01b	0.20 ± 0.01b	0.25 ± 0.02c
Significance	AM	**	**	*	**	**	*	*
	N	**	**	**	**	**	**	**
	AM×N	**	**	**	ns	ns	ns	ns

NAM, non-AMF-inoculated; AM, AMF-inoculated; Different lowercase letters within each column indicate significant differences (*p* < 0.05) among treatments. Values are means ± SE (n = 6). Two-way ANOVA output: ns, not significant; *p < 0.05; **p < 0.01.

Compared with the non-inoculated treatments, the leaf biomass and LMR of mycorrhizal seedlings were higher than those of non-mycorrhizal seedlings under three N concentrations, while the SMR, RMR and root/shoot ratio were lower (**Table 1**). After inoculation with AMF, the stem biomass of mycorrhizal seedlings was slightly lower than that of non-mycorrhizal seedlings at low or high N levels, and significantly increased at medium N levels, while the root biomass decreased slightly at low N levels, and was higher than that of non-inoculated seedlings at medium or high N levels. At medium N levels, the leaf, stem, root biomass and LMR of inoculated seedlings were significantly increased (*p* < 0.05).

### 3.4 Root architecture and activity

N application and AMF inoculation significantly affected the root growth of *C*. *bungei* (**Table 2**; **Supplementary data, Figure S1**). Without inoculation, the root morphological parameters (except the average root diameter) and root activity of seedlings decreased gradually with the increase of N concentration. After inoculation with AMF, all root morphological parameters (except the average root diameter) and root activity of mycorrhizal seedlings showed a trend of first increasing and then decreasing, reaching the maximum at medium N and significantly higher than those at low and high N levels (**Table 2**). In general, the average root diameter of seedlings was not significantly affected by N application regardless of inoculation status. At the same N levels, there were differences in the effects of non-inoculated and inoculated treatments on seedling root growth (**Table 2**; **Supplementary data, Figure S1**). At low N levels, all root morphological parameters (except the average root diameter) and root activity of inoculated seedlings were lower than those of non-inoculated seedlings, and the total root length, length per unit volume, tips, forks, and length of fine roots (0 < d ≤ 0.5 mm) reached significant differences. However, at medium N levels, all parameters (except the average root diameter) and root activity of mycorrhizal seedlings were significantly higher than those of non-mycorrhizal seedlings (*p* < 0.05; 1.24–1.92-fold). At high N levels, there were no significant differences in all root morphological parameters and root activity between inoculated and non-inoculated seedlings.

**Table 2:** Effect of the arbuscular mycorrhizal fungus (AMF) *Rhizophagus intraradices* on the root morphological parameters of *Catalpa bungei* seedlings under different nitrogen (N) concentrations.

NH_4_NO_3_ (mM)	AMF status	Total root length (cm)	Total root surface area (cm^2^)	Root projected area (cm^2^)	Total root volume (cm^3^)	Length per unit volume (cm/m^3^)	Average diameter (mm)	Root tips	Forks	LF (cm)	SAF (cm^2^)	Root activity [μg/(g·h)]
0.25	NAM	1714.77 ± 131.93b	377.85 ± 28.37b	120.27 ± 9.03b	6.72 ± 0.66b	1714.77 ± 131.93b	0.71 ± 0.03	3994.17 ± 341.38b	7786.67 ± 741.03a	1091.15 ± 107.21ab	100.55 ± 10.56b	87.65 ± 5.95ab
	AM	1389.50 ± 86.62c	321.15 ± 20.89b	102.22 ± 6.65b	6.01 ± 0.39b	1389.50 ± 86.62c	0.75 ± 0.01	2546.00 ± 256.13c	5313.67 ± 301.99b	817.33 ± 61.57c	80.46 ± 3.91b	75.82 ± 6.53b
10.00	NAM	1435.35 ± 114.35c	319.41 ± 19.81b	101.67 ± 6.30b	5.74 ± 0.28b	1435.35 ± 114.35c	0.72 ± 0.02	2654.00 ± 426.77c	5457.17 ± 757.11b	877.72 ± 103.37bc	89.72 ± 8.31b	77.03 ± 6.67b
	AM	2108.03 ± 112.42a	468.75 ± 26.97a	149.21 ± 8.59a	8.40 ± 0.67a	2108.03 ± 112.42a	0.71 ± 0.03	5100.67 ± 526.38a	8972.83 ± 738.95a	1265.28 ± 89.89a	127.49 ± 6.97a	95.54 ± 3.18a
45.00	NAM	944.58 ± 37.28d	217.92 ± 9.17c	69.37 ± 2.92c	4.05 ± 0.32c	944.58 ± 37.28d	0.74 ± 0.04	2488.33 ± 191.44c	4414.17 ± 378.15b	571.44 ± 34.83d	54.16 ± 3.23c	52.63 ± 4.02c
	AM	963.25 ± 57.14d	219.16 ± 9.83c	69.76 ± 3.13c	3.98 ± 0.18c	963.25 ± 57.14d	0.73 ± 0.02	2576.50 ± 327.70c	4190.33 ± 457.00b	567.74 ± 42.45d	54.86 ± 3.54c	53.12 ± 3.14c
Significance	AM	ns	ns	ns	ns	ns	ns	ns	ns	ns	ns	ns
	N	**	**	**	**	**	ns	**	**	**	**	**
	AM×N	**	**	**	**	**	ns	**	**	**	**	*

NAM, non-AMF-inoculated; AM, AMF-inoculated; LF, length of fine roots (0 < d ≤ 0.5 mm); SAF, the surface area of fine roots (0 < d ≤ 0.5 mm). Different lowercase letters within each column indicate significant differences (*p* < 0.05) among treatments. Values are means ± SE (n = 6). Two-way ANOVA output: ns, not significant; *p < 0.05; **p < 0.01.

### 3.5 Photosynthetic gas exchange capacity

N application had a certain effect on the photosynthetic gas exchange parameters of seedlings (**Table 3**). Pn and WUE of non-inoculated (or inoculated) seedlings increased significantly at first and then decreased with the increase of N concentration, and reached the maximum at medium N levels. Under non-inoculated conditions, Gs and Tr decreased gradually with the increase of N concentration, while Ci decreased first and then increased slightly. After inoculation with AMF, Gs and Tr of mycorrhizal seedlings increased first and then decreased with the increase of N concentration, while Ci decreased gradually. Under the same N levels, the photosynthetic gas exchange parameters of inoculated seedlings were all higher than those of non-inoculated seedlings (except Ci at high N levels and Tr at low N levels; **Table 3**). At medium N levels, Pn, Gs, Ci and WUE of mycorrhizal seedlings were significantly higher than those of non-mycorrhizal seedlings (*p* < 0.05; 1.10–2.14-fold). However, at low or high N levels, only some indexes were significantly different between inoculated and non-inoculated treatments.

**Table 3:** Effect of the arbuscular mycorrhizal fungus (AMF) *Rhizophagus intraradices* on the photosynthetic gas exchange parameters of *Catalpa bungei* seedlings under different nitrogen (N) concentrations.

NH_4_NO_3_ (mM)	AMF status	P_n_ [μmol/(m^2^·s)]	G_s_ [mol/(m^2^·s)]	C_i_ (µmol/mol)	T_r_ [mmol/(m^2^·s)]	WUE (µmol/mmol)
0.25	NAM	3.98 ± 0.37d	0.22 ± 0.03b	331.46 ± 5.38a	4.90 ± 0.54a	0.94 ± 0.09d
	AM	5.14 ± 0.40cd	0.24 ± 0.03ab	338.51 ± 4.42a	3.10 ± 0.27b	1.72 ± 0.08c
10.00	NAM	6.82 ± 0.45b	0.14 ± 0.02c	295.19 ± 7.13b	3.96 ± 0.41ab	1.86 ± 0.09bc
	AM	8.88 ± 0.32a	0.30 ± 0.02a	324.72 ± 3.93a	4.19 ± 0.27ab	2.24 ± 0.13a
45.00	NAM	5.34 ± 0.48c	0.10 ± 0.01c	298.97 ± 6.07b	3.16 ± 0.31b	1.76 ± 0.09c
	AM	7.03 ± 0.55b	0.14 ± 0.02c	270.28 ± 7.23c	3.45 ± 0.30b	2.09 ± 0.08ab
Significance	AM	**	**	ns	ns	**
	N	**	**	**	ns	**
	AM×N	ns	**	**	**	*

NAM, non-AMF-inoculated; AM, AMF-inoculated; Pn, net photosynthetic rate; Gs, stomatal conductance; Tr, transpiration rate; Ci, intercellular CO_2_ concentration; WUE, water use efficiency. Different lowercase letters within each column indicate significant differences (*p* < 0.05) among treatments. Values are means ± SE (n = 18). Two-way ANOVA output: ns, not significant; *p < 0.05; **p < 0.01.

### 3.6 Nutrient uptake and distribution

**Figure 3:**
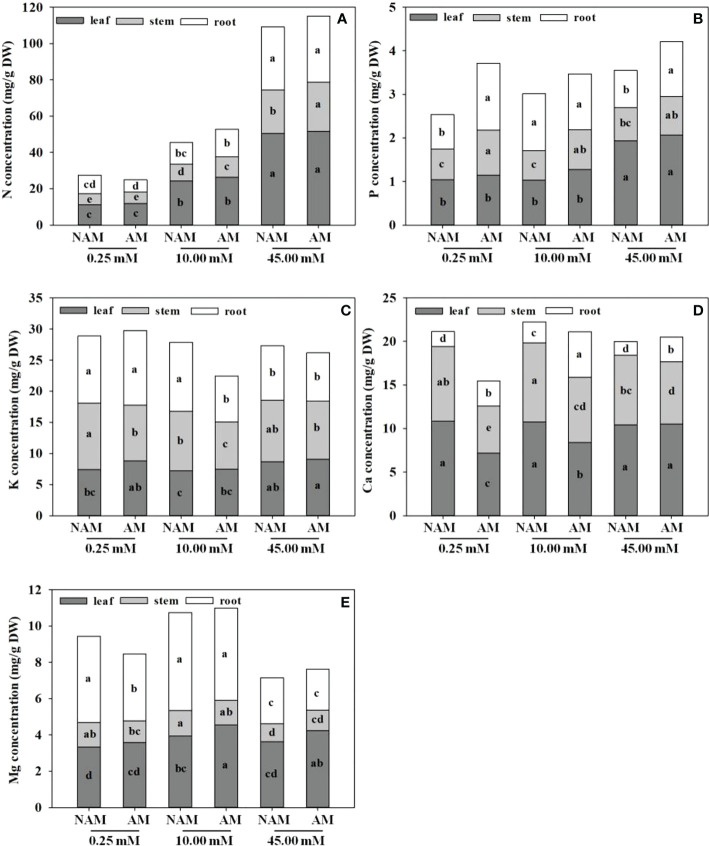
Effect of the arbuscular mycorrhizal fungus (AMF) *Rhizophagus intraradices* on the **(A)** nitrogen (N), **(B)** phosphorus (P), **(C)** potassium (K), **(D)** calcium (Ca) and **(E)** magnesium (Mg) concentrations of *Catalpa bungei* seedlings under different nitrogen (N) levels. NAM, non-AMF-inoculated; AM, AMF-inoculated. Different lowercase letters above the bars indicate significant differences (*p* < 0.05) among treatments. Values are means ± SE (*n* = 6).

In general, the N concentration was the highest in *C*. *bungei* seedlings, followed by K, Ca, and Mg concentrations, while P concentration was the lowest (**Figure 3**). No matter whether inoculated or not, or the level of N application, the accumulation of N concentration was highest in leaves, followed by roots, and lowest in stems (**Figure 3A**). P and Mg concentrations were higher in leaves and roots, but lowest in stems (**Figures 3B, E**). Ca concentration was highest in leaves, followed by stems and lowest in roots (**Figure 3D**). At low to medium N levels, K concentration in different parts of inoculated (or non-inoculated) seedlings was ranked as root > stem > leaf, while at high N levels, K concentration was the highest in the stem, followed by leaf and root (**Figure 3C**).

Regardless of AMF inoculation status, with the increase of N application, the N concentration in leaves, stems and roots of *C*. *bungei* seedlings significantly increased, and the K concentration first decreased and then increased (except the K concentration in roots without inoculation). On the contrary, the Ca and Mg concentrations in seedlings showed a trend of first increasing and then decreasing (except the Ca concentration in leaves), reaching the maximum at medium N levels (**Figure 3**). The P concentration in leaves of both inoculated and non-inoculated seedlings increased with the increase of N application, while P concentration in stems didn’t change significantly. The P concentration in roots increased significantly at first and then decreased markedly with the increase of N application without inoculation (*p* < 0.05), while there was no significant change under inoculation conditions. With the increase of N application, the Ca concentration in leaves didn’t change notably under non-inoculated conditions, but increased significantly under inoculation. At the same N levels, the N and P concentrations in leaves, stems and roots, K and Mg concentrations in leaves, and Ca concentrations in roots of mycorrhizal seedlings were higher than those of non-mycorrhizal seedlings (except N concentration in roots under low N condition), while the K and Mg concentrations in stems and roots and Ca concentrations in leaves and stems were lower (except K concentration in roots under low N levels and Mg concentration in stems under high N levels; **Figure 3**).

### 3.7 Phytohormones

**Figure 4:**
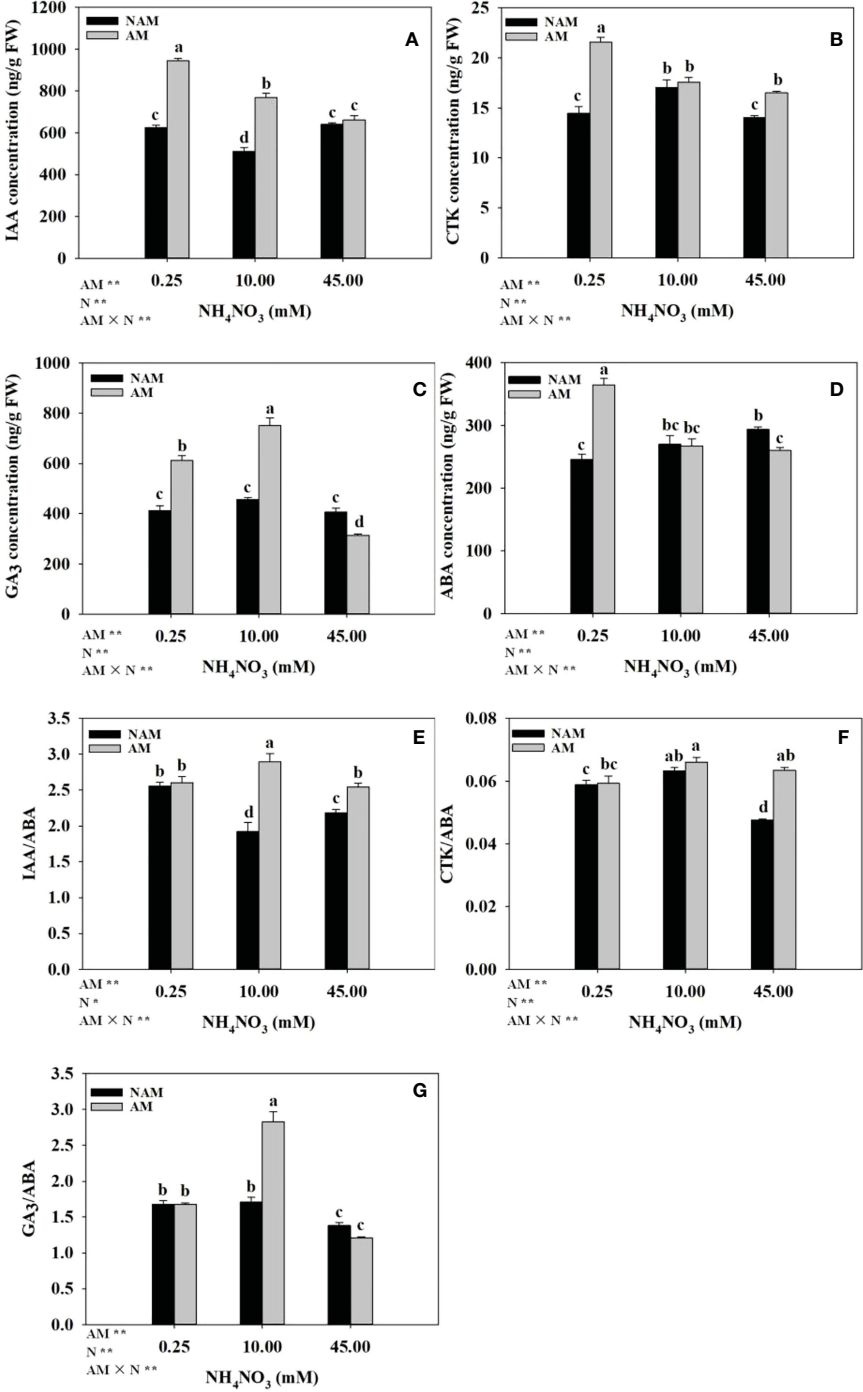
Effect of the arbuscular mycorrhizal fungus (AMF) *Rhizophagus intraradices* on the phytohormones concentrations and ratios in the root system of *Catalpa bungei* seedlings under different nitrogen (N) levels. **(A)**, indole-3-acetic acid (IAA); **(B)**, cytokinin (CTK); **(C)**, gibberellin (GA_3_); **(D)**, abscisic acid (ABA); **(E)**, IAA/ABA; **(F)**, CTK/ABA; **(G)**, GA_3_/ABA. NAM, non-AMF-inoculated; AM, AMF-inoculated. Different lowercase letters above the bars indicate significant differences (*p* < 0.05) among treatments. Values are means ± SE (*n* = 6). Two-way ANOVA output: ns, not significant; *p < 0.05; **p < 0.01.

Both N application and AMF inoculation significantly changed the hormone concentrations and ratios in the root system of seedlings (**Figure 4**). Without AMF inoculation, with the increase of N application level, IAA concentration and IAA/ABA ratio in roots showed a trend of first decreasing and then increasing, ABA concentration gradually increased, while CTK, GA_3_ concentrations, and CTK/ABA, GA_3_/ABA ratios increased first and then decreased. After AMF inoculation, the concentrations of IAA, CTK and ABA in roots of mycorrhizal seedlings decreased gradually with the increase of N concentration, while the GA_3_ concentration and the ratios of hormones (IAA/ABA, CTK/ABA, GA_3_/ABA) showed a trend of increasing first and then decreasing, and the concentrations of IAA, GA_3_ and the ratio of GA_3_/ABA were significantly different among the three N concentrations (*p* < 0.05). Under the same N levels, the root hormone concentrations (IAA, CTK, GA_3_) and ratios (IAA/ABA, CTK/ABA, GA_3_/ABA) of the inoculated seedlings were higher than those of non-inoculated treatments (except GA_3_ content and GA_3_/ABA ratio at high N levels), while the ABA concentration was lower (except ABA content at low N levels; **Figure 4**). At low N levels, the four hormones (IAA, CTK, GA_3_, ABA) of inoculated treatments were about 1.50-fold of those of non-inoculated treatments. At medium N levels, the differences in IAA, GA_3_ concentrations, and IAA/ABA, GA_3_/ABA ratios between inoculated and non-inoculated treatments were significant, which were 1.50–1.66-fold of those of non-inoculated treatments, respectively.

### 3.8 Activities of key enzymes for N metabolism in roots

**Figure 5:**
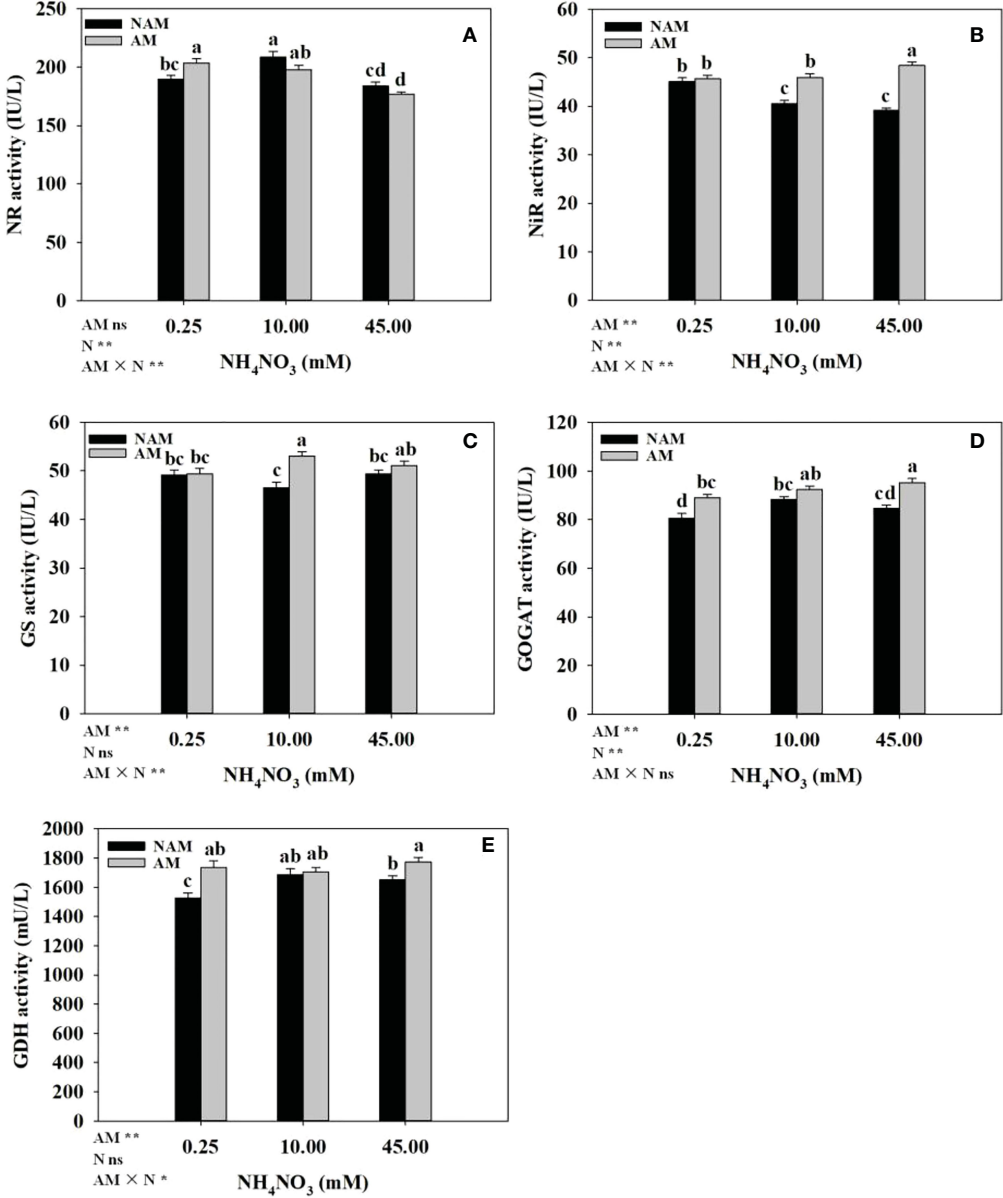
Effect of the arbuscular mycorrhizal fungus (AMF) *Rhizophagus intraradices* on the activities of key enzymes of nitrogen (N) metabolism in the root system of *Catalpa bungei* seedlings under different N levels. **(A)**, nitrate reductase (NR); **(B)**, nitrite reductase (NiR); **(C)**, glutamine synthetase (GS); **(D)**, glutamate synthetase (GOGAT); **(E)**, glutamate dehydrogenase (GDH). NAM, non-AMF-inoculated; AM, AMF-inoculated. Different lowercase letters above the bars indicate significant differences (*p* < 0.05) among treatments. Values are means ± SE (*n* = 7). Two-way ANOVA output: ns, not significant; *p < 0.05; **p < 0.01.

The effects of N application on the activities of key enzymes of N metabolism in the roots of inoculated and non-inoculated seedlings were different (**Figure 5**). Without inoculation, the activities of NR, GOGAT and GDH in the roots increased significantly at first with the increase of N concentration, reached the maximum at medium N levels, and then decreased (**Figures 5A, D, E**). The NiR activity decreased gradually (**Figure 5B**), while GS activity didn’t change significantly (**Figure 5C**). After AMF inoculation, with the increase of N concentration, NR activity in the roots gradually decreased (**Figure 5A**), NiR and GOGAT activities gradually increased (**Figures 5B, D**), and GS activity first significantly increased and then slightly decreased (**Figure 5C**), while the GDH activity didn’t change much (**Figure 5E**). At the same N levels, the activities of key enzymes (NR, NiR, GS, GOGAT, GDH) in the roots of inoculated seedlings were higher than those of non-inoculated seedlings (except NR activity at 10–45 mM; **Figure 5**). Among them, the activities of NR, GOGAT, GDH at low N levels, NiR, GS at medium N levels, and NiR, GOGAT, GDH at high N levels were significantly different between inoculated and non-inoculated treatments.

### 3.9 Relative expression of genes related to N metabolism in roots

**Figure 6:**
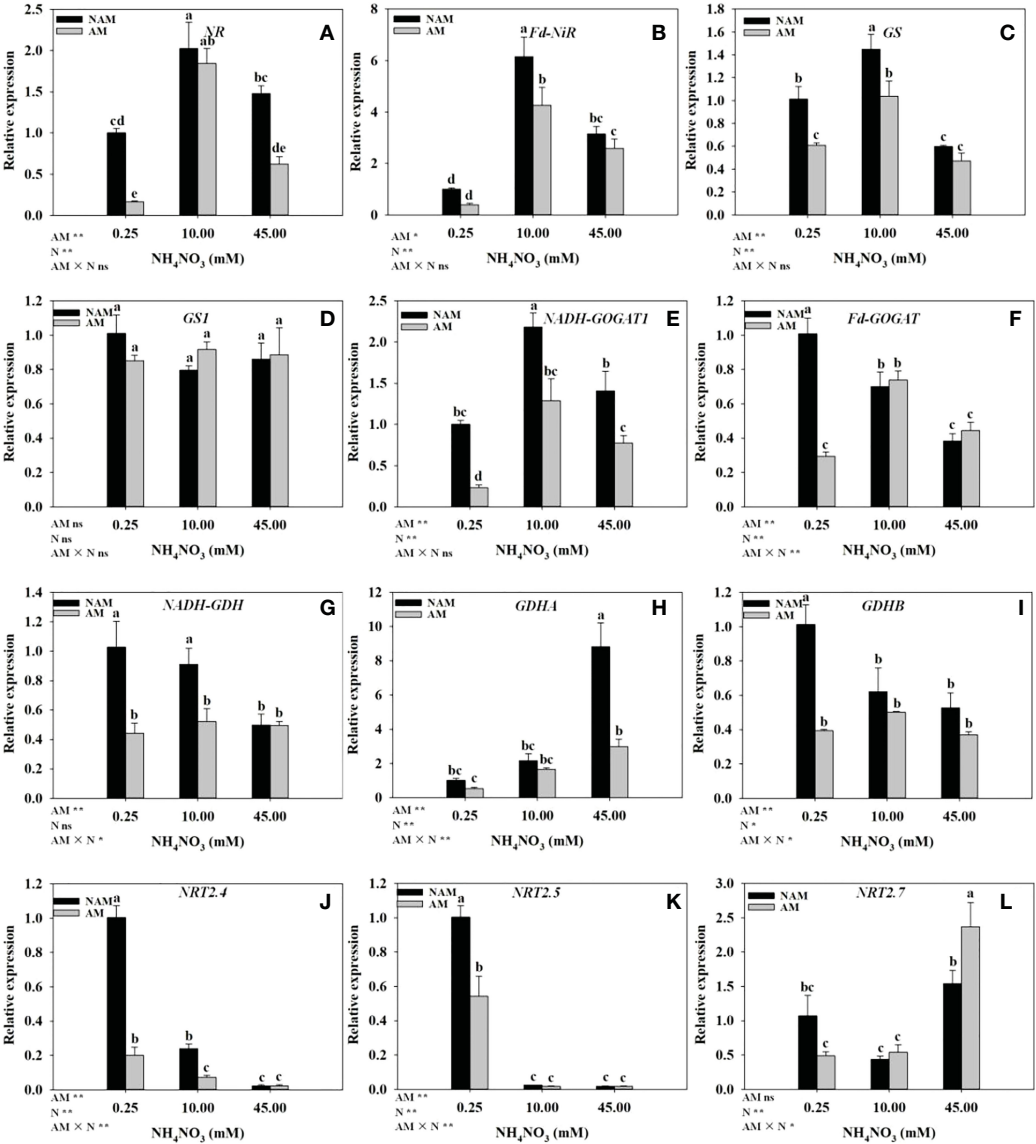
Effect of the arbuscular mycorrhizal fungus (AMF) *Rhizophagus intraradices* on the relative expression of genes related to nitrogen (N) metabolism in the root system of *Catalpa bungei* seedlings under different N levels. **(A)**, nitrate reductase (*NR*); **(B)**, ferredoxin-nitrite reductase (*Fd-NiR*); **(C)**, glutamine synthetase (*GS*); **(D)**, glutamine synthetase cytosolic isozyme 2 (*GS1*); **(E)**, glutamate synthase 1[NADH] (*NADH-GOGAT1*); **(F)**, ferredoxin-dependent glutamate synthase (*Fd-GOGAT*); **(G)**, NADP-specific glutamate dehydrogenase (*NADH-GDH*); **(H)**, glutamate dehydrogenase A (*GDHA*); **(I)**, glutamate dehydrogenase B (*GDHB*); **(J)**, high affinity nitrate transporter 2.4-like (*NRT2.4*); **(K)**, high affinity nitrate transporter 2.5 (*NRT2.5*); **(L)**, high affinity nitrate transporter 2.7-like (*NRT2.7*). NAM, non-AMF-inoculated; AM, AMF-inoculated. Different lowercase letters above the bars indicate significant differences (*p* < 0.05) among treatments. Values are means ± SE (*n* = 3). Two-way ANOVA output: ns, not significant; *p < 0.05; **p < 0.01.

N application significantly affected the expression levels of genes related to N metabolism in seedling roots (**Figure 6**). Regardless of AMF inoculation status, with the increase of N concentration, the relative expression of *NR*, *Fd-NiR* and *GS* genes in the roots of *C*. *bungei* seedlings were significantly up-regulated at first and then markedly down-regulated (**Figures 6A**–C), while the relative expression of *GS1* had no significant change (**Figure 6D**). Without inoculation, with the increase of N concentration, the relative expression of *NADH-GOGAT1* in roots was firstly significantly up-regulated and then markedly down-regulated (**Figure 6E**). On the contrary, the relative expression of *NRT2.7* was first down-regulated and then up-regulated (**Figure 6L**), while the expression of *GDHA* was gradually up-regulated (**Figure 6H**), and the relative expression of *Fd-GOGAT*, *NADH-GDH*, *GDHB*, *NRT2.4*, and *NRT2.5* were gradually down-regulated (**Figures 6F, G, I–K**), and *Fd-GOGAT*, *NRT2.4* were significantly different among the three N levels (*p* < 0.05). After AMF inoculation, the relative expression of *NADH-GOGAT1*, *Fd-GOGAT*, *NADH-GDH*, and *GDHB* in roots were firstly up-regulated and then down-regulated with the increase of N concentration, reaching the maximum at medium N levels (**Figures 6E–G, I**), and the relative expression of *GDHA*, *NRT2.7* was gradually up-regulated (**Figures 6H, L**). However, the relative expression of *NRT2.4* and *NRT2.5* was gradually down-regulated (**Figures 6J, K**). At the same N levels, compared with the non-inoculated treatment, the relative expression of *NR*, *Fd-NiR*, *GS*, *NADH-GOGAT1*, *NADH-GDH*, *GDHA*, *GDHB*, *NRT2.4*, and *NRT2.5* in roots of mycorrhizal seedlings were all down-regulated (**Figures 6A–C, E, G–K**). In addition, at low to medium N levels, the relative expression of most of the above genes reached significant differences between inoculated and non-inoculated treatments. At low N levels, the relative expression of *GS1*, *Fd-GOGAT* and *NRT2.7* in roots of inoculated seedlings was lower than those of non-inoculated seedlings, while at medium to high N levels, the expression of the above three genes was higher (**Figures 6D, F, L**).

### 3.10 PCA of growth and physiological responses to N application and inoculation

**Figure 7:**
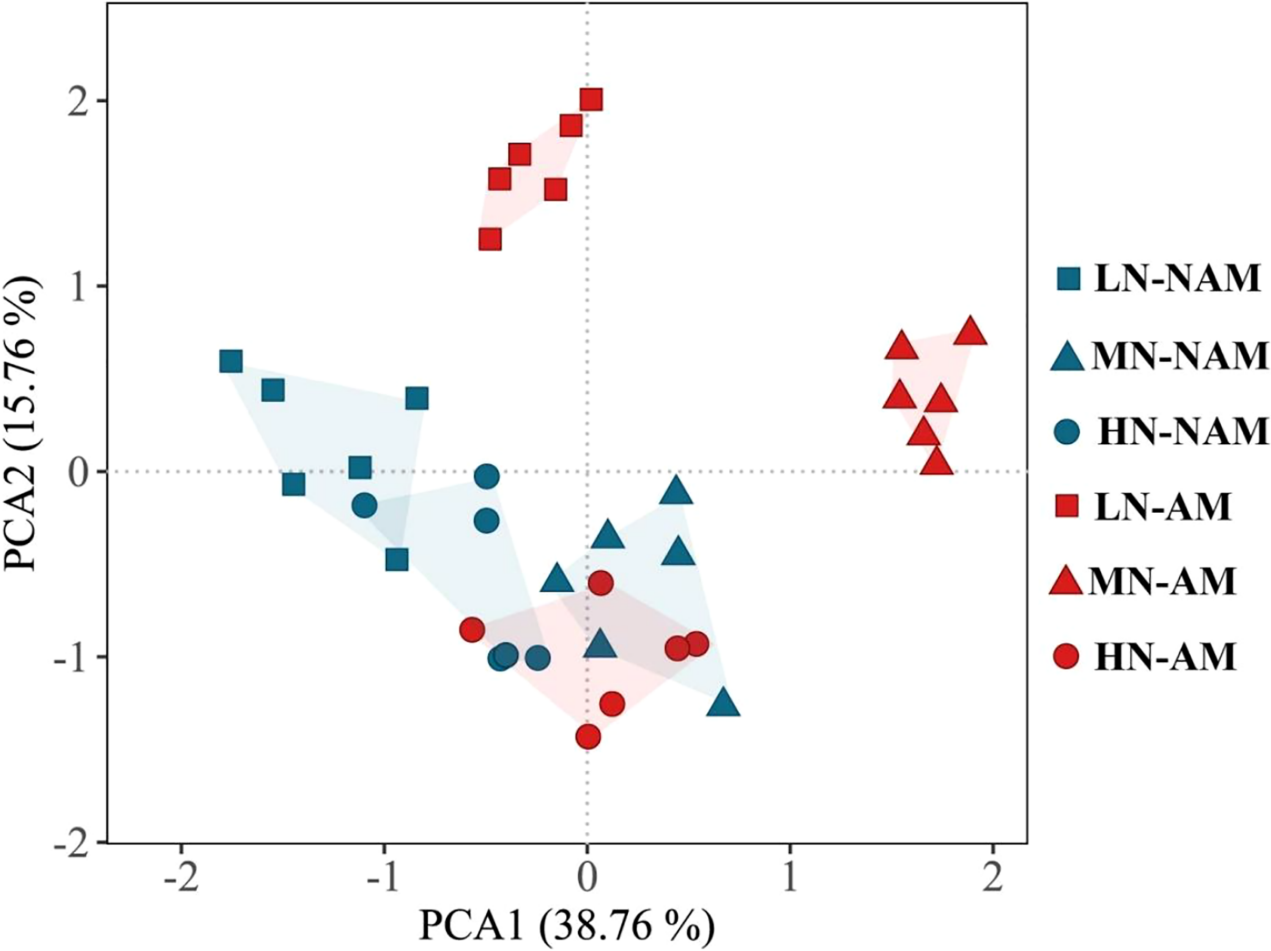
Principal component analysis (PCA) plot of growth and physiological characteristics of *Catalpa bungei* under different nitrogen (N) levels and inoculation. LN, low N level (0.25 mM); MN, moderate N level (10 mM); HN, high N level (45 mM); NAM, non-AMF-inoculated; AM, AMF-inoculated.

To reveal the key parameters involved in the response patterns of *C*. *bungei* to N supply levels or inoculation of AMF, PCA was performed using data of morphological and physiological parameters related to growth, photosynthesis, root hormones, and activities of key enzymes of N metabolism (**Figure 7**; **Supplementary data, Table S2**). PC1 and PC2 accounted for 38.76% and 15.76% of the variation, respectively. PC1 distinguished the change in the N effect under non-inoculated treatments, while PC2 clearly revealed the effect of N treatment levels after AMF inoculation. Plant height, total biomass, leaf area, photosynthetic pigments, Pn, Fv/Fm and Fv/Fo were key contributors to PC1, while gas exchange parameters (Gs, Ci), and root hormones (IAA, CTK, GA_3_, ABA) were important factors to PC2 (**Supplementary data, Table S2**). In the PCA plot, the greater the distance between the symbols associated with the N treatment levels, the stronger the response of morphological and physiological parameters to changes in N supply levels. After AMF inoculation, the distance between the symbols associated with different N levels was larger than that of non-inoculation treatments, indicating that the inoculation was more sensitive to the change of N supply levels. These results suggested that *C*. *bungei* seedlings inoculated with and without AMF exhibit distinct morphological and physiological responses in response to N availability.

## 4 Discussion

### 4.1 Mycorrhizal colonization and seedling growth

The mycorrhizal colonization rate is an important indicator of close symbiosis (Wang et al., 2021b), which reflects the affinity between fungi and host plants (Li et al., 2022). In general, the total colonization rates under three N levels were higher than 65%, indicating that the root system of *C*. *bungei* could form a good symbiotic system with *R*. *intraradices*. Studies have shown that AMF are very sensitive to the nutrient status of their environment (Kong et al., 2022). Our study found that moderate N addition could significantly increase the colonization rate of *R*. *intraradices* and promote the growth of *C*. *bungei* seedlings, but excessive N could inhibit mycorrhizal colonization and the growth of seedlings, which was similar to the previous results in *Camellia sinensis* (Zhao et al., 2014), *Sophora japonica* (Yang et al., 2021). Several studies have shown that mycorrhizal symbiosis could promote the growth of woody plants (Turjaman et al., 2006; Outamamat et al., 2022; Tomazelli et al., 2022). However, the effects of AMF on plant growth can be altered by soil N levels. Wu et al. (2017b) showed that inoculation of *R*. *irregularis* at low N levels significantly promoted the growth of *Populus × canadensis*. In the present work, inoculation with AMF at medium N levels significantly promoted the growth parameters (plant height, basal diameter and total biomass), and significant positive correlations (*p* < 0.01) were found between the percentage of mycorrhizal colonization and growth parameters (**Supplementary data, Table S3**), which also confirmed the positive role of AMF. It could be attributed to the fact that AMF promoted the absorption of nutrients and water in seedlings (Yang et al., 2014). However, when the N concentration was too low or too high, the promoting effect of AMF on *C*. *bungei* seedlings decreased. Hawkins et al. (2000) reported that under different N concentrations, inoculation of *Glomus mosseae* had no significant effect on the growth of *Triticum aestivum*. However, studies on *Eucalyptus urophylla* (Qin and Yu, 2019) and *P*. *euramericana* (Rooney et al., 2011) showed that the growth of plants was inhibited after inoculation with AMF. These inconsistent results indicate that in addition to the nutrient supply level of the plant environment, there are also factors such as fungi, host plant species, and the coordination between fungi and hosts, which jointly determine whether AMF function may be effectively performed (Evangelina et al., 2015; Qin and Yu, 2019).

### 4.2 Biomass allocation

In the process of plant growth, the balance of biomass allocation between underground and aboveground is the key to plant growth, which greatly affects the adaptability and competitiveness of plants (Zhang et al., 2021). In this study, whether inoculated or not, the SMR of *C*. *bungei* seedlings increased first and then decreased with the increase of N concentration, and the LMR increased gradually, while RMR and root/shoot ratio decreased gradually. These were consistent with the results of N application on biomass allocation in *Acer mono* (Xiao et al., 2015) and *Carya illinoensis* (Wang et al., 2018). The possible explanation is that when the available N supply is insufficient, the carbon content allocated by plants to roots is higher, which is conducive to the absorption of more N by roots. However, when the supply of N is relatively sufficient, the proportion of photosynthate allocated to the aboveground part increases, which is helpful to improve the ability to transform light energy and promote the accumulation of photosynthate (Hermans et al., 2006; Meng et al., 2022). After inoculation with AMF, compared with non-mycorrhizal seedlings, mycorrhizal *C*. *bungei* seedlings significantly increased the proportion of plant biomass allocated to leaves, but decreased the proportion allocated to stems and roots, thus changing the biomass allocation. Indeed, due to the establishment of AMF, the alleviation of nutrient restriction of host plants may lead to the reduction of root/shoot ratio, which is also confirmed by our results that the percentage of mycorrhizal colonization was negatively correlated with RMR and root/shoot ratio (**Supplementary data, Table S3**). Alternatively, woody plants tend to distribute aboveground and underground biomass more unevenly in favor of aboveground growth, and may reflect the ability of AMF to further promote plant growth (Veresoglou et al., 2012). Similar results were also obtained in mycorrhizal *C*. *sinensis* (Singh et al., 2010).

### 4.3 Photosynthesis

Photosynthesis is an indispensable part of the life activities of green plants, which can provide the necessary energy source for the metabolism of almost all organisms (Yang et al., 2014; Sun et al., 2021). Our study indicated that moderate N concentration could enhance photosynthetic gas exchange parameters (Pn, WUE), photosynthetic pigment contents (**Supplementary data, Table S4**), leaf area and SLA (**Supplementary data, Figures S2, S3**), and chlorophyll fluorescence parameters (except NPQ; Supplementary data, Table S5) of both mycorrhizal and non-mycorrhizal *C*. *bungei* seedlings. When the N concentration was too low or too high, photosynthesis was inhibited. These results were in accordance with the findings in *Handroanthus heptaphyllus* (Berghetti et al., 2021) and *Cyclocarya paliurus* (Qin et al., 2021). It was suggested that within a certain range, the application of N can increase the leaf area and SLA of plants, enhance the efficiency of capturing solar radiation, and thus enhancing photosynthesis (Seepaul et al., 2016). Mycorrhizal symbiosis also affects photosynthesis (Augé, 2000). After colonizing roots, AMF could maintain the high gas exchange capacity of plants and enhance photosynthesis, which has also been documented in mycorrhizal *Robinia pseudoacacia* (Zhu et al., 2014), *Gleditsia sinensis* (Wang et al., 2019) and *Ilex paraguariensis* (Tomazelli et al., 2022). In particular, in most cases, we also recorded a significant positive correlation between the percentage of mycorrhizal colonization and photosynthetic characteristic parameters (*p* < 0.01, or 0.05; **Supplementary data, Table S3**). In addition, under the same N levels, the photosynthetic gas exchange parameters (except Tr at low N and Ci at high N), photosynthetic pigment contents (**Supplementary data, Table S4**), leaf area, SLA (**Supplementary data, Figures S2, S3**), and chlorophyll fluorescence parameters (**Supplementary data, Table S5**) of mycorrhizal seedlings were all higher than those of non-inoculated seedlings, and most of them reached significant differences between the inoculated and non-inoculated treatments at low to medium N levels. These results indicated that AMF promoted the photosynthesis of *C*. *bungei* seedlings, and the effect was more obvious in a moderate level range. On the one hand, this may be related to the promotion of P and Mg uptake by AMF (Zhu et al., 2014; Outamamat et al., 2022). In our study, the results that the percentage of mycorrhizal colonization was positively correlated with leaf Mg concentration (*p* < 0.01, or 0.05) also confirmed the above hypothesis (**Supplementary data, Table S3**). On the other hand, mycorrhizal plants with higher SLA tended to increase the allocation of leaf N in the photosynthetic system, and had higher photosynthetic capacity and N utilization efficiency (Wu et al., 2017a; Lu et al., 2021).

### 4.4 Nutrient absorption and distribution

Some studies suggested that in the process of nutrient supply, seedlings would successively be in the three stages of nutrient deficiency, luxury consumption and nutrient toxicity (Timmer, 1997; Salifu and Timmer, 2003). In particular, during the nutrition toxicity stage, the N content still increased with the increase of N application, while the biomass even declined. In the present study, regardless of AMF inoculation status, the N concentration in the leaves, stems and roots of *C*. *bungei* seedlings increased significantly with the increase of N application, and the N concentration at high N levels was about 3.5–5-fold that at low N levels (**Figure 3**), but the growth at high N levels was significantly inhibited (**Figure 2**), which also confirmed the above conclusions. In general, moderate N levels could promote the absorption and accumulation of nutrient elements (N, P, Ca, Mg) in different parts of *C*. *bungei* seedlings, and similar conclusions have been obtained in other woody plants (Wu et al., 2017b; Qin et al., 2021). Several studies have reported that AMF contribute to the uptake and accumulation of nutrients (P, N, K) in a variety of woody plants under certain conditions (Turjaman et al., 2006; Ouahmane et al., 2007; Outamamat et al., 2022). This may be due to the fact that AMF hyphae are much thinner than plant roots, which can penetrate smaller pores and expand root uptake area, thus improving the plant’s access to nutrients, especially those with poor ionic mobility or lower concentrations in soil solutions (Begum et al., 2019; Wang et al., 2019). In the current study, the results showed that AMF inoculation could promote the accumulation of nutrient elements (especially N and P) in seedlings, change the distribution of nutrient elements in different parts, and improve the growth of seedlings at different N levels. Evidence suggests that N and P are closely related to plant photosynthesis. P is an important component of enzymes required for plant photosynthesis and ATP (Shan et al., 2020). While N is the main component of chlorophyll, about 60% of the total N in leaves exists in chloroplasts, which can regulate the activities of enzymes related to the photosynthetic electron transport chain (Berghetti et al., 2021; Qin et al., 2021), and the increase of plant N content within a certain range would promote photosynthesis (Berghetti et al., 2021). Therefore, the promotion of symbiotic plant growth by AMF is closely related to the improvement of plant nutrition and the enhancement of plant photosynthesis, which was in accordance with the results of this study.

### 4.5 Root architecture and hormone levels

The root system is an important organ of plants, which participates in the acquisition and storage of water and nutrients (Khan et al., 2021). It is generally believed that a low N level can stimulate the root development of woody plants, while a high N level can inhibit root growth (Luo et al., 2013; Song et al., 2019). In this study, low N could significantly stimulate root morphological parameters and root activity of *C*. *bungei* seedlings without inoculation. However, after inoculation with AMF, moderate N addition significantly promoted the development of mycorrhizal seedling roots. When N was excessive, the growth of seedling roots could be significantly inhibited regardless of inoculation status. These results were in keeping with those of previous studies. Several studies have shown that AMF colonization can improve the root architecture of woody plants, and directly affect the uptake of soil water and mineral nutrients by plants (Zhang et al., 2016; Gao et al., 2019; Tomazelli et al., 2022). In our study, compared with non-inoculated treatment, AMF inoculation could significantly improve root architecture, enhance root activity, and stimulate root growth of *C*. *bungei* seedlings under moderate N concentration, while too low or too high N concentrations were not conducive to mycorrhizal effect.

The change of hormone balance in root tissue may be related to the change in root morphology (Hermans et al., 2006). Although auxin is considered to be the major hormone regulating the function of the root meristem, ABA and GA_3_ have been proved to play an important role in regulating lateral root formation, such as cell division and cell elongation (Band et al., 2012; Harris, 2015). In the current study, root ABA concentration was negatively correlated with root morphological parameters (except the average diameter), and reached a significant difference with root tips and forks (*p* < 0.05; **Supplementary data, Table S6**), indicating that ABA level negatively regulated the root development of *C*. *bungei*. In *Arabidopsis thaliana*, GA_3_ could positively control the growth of root apical meristem and the formation of lateral roots (Achard et al., 2009; Ubeda-Tomás et al., 2009). In particular, in our study, GA_3_ concentration was significantly positively correlated with root morphological parameters (except the average diameter) and root activity (*r* was in the range of 0.410–0.657, *p* < 0.01, or 0.05; **Supplementary data, Table S6**), indicating that GA_3_ could significantly promote the growth and development of roots of *C*. *bungei* seedlings. However, in *Medicago truncatula* (Fonouni-Farde et al., 2019) and *Populus* (Gou et al., 2010), it was found that GA_3_ had a negative impact on root growth. These results suggested that the regulation of root structure by phytohormones varies among plant species. In addition, ABA can also exert at least partial effects on root growth by crosstalk with hormone signals such as GA_3_ and IAA (Gou et al., 2010; Harris, 2015). In the present study, the ratios of IAA/ABA, CTK/ABA, and GA_3_/ABA were significantly positively correlated with most of the root morphological parameters except the average diameter (*p* < 0.01, or 0.05; **Supplementary data, Table S6**), which also confirmed the above conclusions.

### 4.6 Activities of key enzymes and expression of genes in N metabolism

Within a certain concentration range, N application could increase the activity of N metabolizing enzymes (NR, NiR, GS, GOGAT, GDH) and up-regulate the expression levels of related genes in woody plant seedlings (Luo et al., 2013; Qin et al., 2021; Sun et al., 2021). In our study, regardless of AMF inoculation status, with the increase of N concentration, the relative expression levels of *NR*, *Fd-NiR*, and *GS* in the roots of *C*. *bungei* seedlings were significantly up-regulated at first and then notably down-regulated, the expression of *GDHA* was gradually up-regulated. However, with the increase of N concentration, the enzyme activities and the expression trend of other genes were not consistent between mycorrhizal and non-mycorrhizal seedlings. It indicated that, on the one hand, there were differences in N metabolism between mycorrhizal and non-mycorrhizal *C*. *bungei* seedlings in response to N application. On the other hand, moderate N levels could effectively improve the process of N metabolism. Evidence suggests that AMF can induce genes and enzyme activities related to nutrient transport and assimilation to help host plants absorb and utilize more N and P (Khan et al., 2021; Ren et al., 2022). It is worth noting that in our study, within a certain range of N concentration, AMF inoculation enhanced the activities of key enzymes of N metabolism in the roots of *C*. *bungei* seedlings, increased plant N concentration, but unexpectedly, the expression levels of most related enzyme genes decreased. The discordance between the enzyme activity and the expression of related genes has been similarly reported in *Oryza sativa* (Cao et al., 2008) and *Zea mays* (Kaldorf et al., 1998). Possible explanations are that, on the one hand, changes in enzyme activity may be the result of integrated regulation by multiple genes (Cao et al., 2008). On the other hand, it is also possible that after mycorrhizal formation in plants, the absorption and transport of N by the mycelial pathway dominated, while the direct root absorption pathway was inhibited (Duan et al., 2016), and the absorption of P by arbuscular mycorrhizal pathway also existed in a similar situation (Smith and Smith, 2011). It has been proved that arbuscules have metabolic activity, especially in plant nutrient exchange and plant respiration (Cox and Tinker, 1976; Kaldorf et al., 1998).

After N addition, the expression of high-affinity transporters *NRT2.1*, *NRT2.2*, and *NRT2.3* in *T*. *aestivum* roots decreased (Duan et al., 2015), which was in accordance with our result that *NRT2.4* and *NRT2.5* in the roots of *C*. *bungei* seedlings down-regulated sharply with the increase of N levels. This is not unexpected, since high-affinity NRT genes are dominant in regulating N uptake at low exogenous ${\text{NO}}_{3}^{-}$ concentrations (Pérez-Tienda et al., 2014; Duan et al., 2015). The expression of NRT in plants involved in the direct N absorption pathway may also be regulated by AMF colonization in host plants (Duan et al., 2015). In our study, under the same N levels, the relative expression levels of *NRT2.4* and *NRT2.5* in the roots of seedlings inoculated with AMF down-regulated compared with those of non-inoculated treatments. Duan et al. (2015) reported that the expressions of *NRT1.2*, *NRT2.1*, *NRT2.2* and *NRT2.3* in *T*. *aestivum* roots, except *NRT1.1*, may be locally down-regulated by specific AMF, regardless of whether N was applied. This is in keeping with the results of this study, and also supports the hypothesis that mycorrhizal symbiosis may reduce direct pathway N uptake. In addition, in the present study, the expression of *NRT2.7* was higher at medium to high N levels after AMF inoculation than that in the non-inoculated treatments. Previous studies have also found that *NRT2.3* expression was up-regulated in *Lycopersicon esculentum* after *G*. *intraradices* inoculation (Hildebrandt et al., 2002). It can be speculated that the regulation of AMF inoculation on the expression of NRT genes in plant roots varies with plant species and gene types (Duan et al., 2016).

## 5 Conclusions

Medium N could significantly improve the physiological metabolism and growth of *C*. *bungei* seedlings. Compared with the non-inoculated treatments, inoculation with *R*. *intraradices* could significantly improve photosynthesis, promote the absorption of N and P, and change root architecture and hormone levels under low to medium N levels. In particular, under N addition, AMF inoculation could improve the absorption and assimilation of N in seedlings by regulating the expression levels of key enzyme genes of N metabolism and nitrate transporter genes in roots, and enhancing the activities of the key enzyme. We preliminarily concluded that *R*. *intraradices* could improve the growth and performance of *C*. *bungei*, and moderate N levels contribute to the beneficial effects of this mycorrhizal fungus. However, only one fungus was used in this study, but different fungal species may perform different levels of benefits to plants under variable environmental conditions. Whether the results of this study could be extrapolated to other species of mycorrhizal fungi and/or to *C. bungei* growing in the field still needs further study.

## Data availability statement

The original contributions presented in the study are publicly available. This data can be found here: https://www.ncbi.nlm.nih.gov/bioproject/under the accession number PRJNA907402.

## Author contributions

Conceptualization, CW and WC; Formal analysis, WC, XM, PM, and JC; Funding acquisition, YZ and CW; Methodology, WC, XM, JC, PM, XT, GM, and KX; Supervision, CW; Writing–Original Draft Preparation, WC; Writing–Review and Editing, WC, YZ, and CW. All authors contributed to the article and approved the submitted version.

## References

[B1] Achard P. Gusti A. Cheminant S. Alioua M. Dhondt S. Coppens F.. (2009). Gibberellin signaling controls cell proliferation rate in *Arabidopsis* . Curr. Biol. 19, 1188–1193. doi: 10.1016/j.cub.2009.05.059 19576768

[B2] Augé R. M. (2000). “Stomatal behavior of arbuscular mycorrhizal plants,” in Arbuscular mycorrhizas (physiology and function). (Dordrecht: Springer). doi: 10.1007/978-94-017-0776-3_10

[B3] Band L. R. Úbeda-Tomás S. Dyson R. J. Middleton A. M. Hodgman T. C. Owen M. R.. (2012). Growth-induced hormone dilution can explain the dynamics of plant root cell elongation. PNAS 109, 7577–7582. doi: 10.1073/pnas.1113632109 22523244 PMC3358831

[B4] Bao S. D. (2000). Soil agrochemical analysis (3rd ed.). China Agric. press Beijing China, 264–270.

[B5] Begum N. Qin C. Ahanger M. A. Raza S. Khan M. I. Ashraf M.. (2019). Role of arbuscular mycorrhizal fungi in plant growth regulation: implications in abiotic stress tolerance. Front. Plant Sci. 10. doi: 10.3389/fpls.2019.01068 PMC6761482 31608075

[B6] Berghetti Á.L.P. Araujo M. M. Tabaldi L. A. Turchetto F. Tassinari A. Bernardy D.. (2021). Effects of nitrogen fertilization on the growth and on photochemical efficiency in plants of *Handroanthus heptaphyllus* . J. Plant Nutr. 44, 2464–2475. doi: 10.1080/01904167.2021.1899216

[B7] Cao Y. Fan X. R. Sun S. B. Xu G. H. Hu J. Shen Q. R. (2008). Effect of nitrate on activities and transcript levels of nitrate reductase and glutamine synthetase in rice. Pedosphere 18, 664–673. doi: 10.1016/S1002-0160(08)60061-2

[B8] Chen H. L. Huang G. W. Ma L. J. Zhang X. Y. Fan X. P. Rong X. J. (2021). Observation on phenology and growth rhythms of six *Catalpa* clones. J. Northeast For. Univ. 49, 11–17. doi: 10.13759/j.cnki.dlxb.2021.08.003

[B9] Chen W. Meng P. Feng H. Wang C. (2020). Effects of arbuscular mycorrhizal fungi on growth and physiological performance of *Catalpa bungei* C.A.Mey. under drought stress. Forests 11, 1117. doi: 10.3390/f11101117

[B10] Cox G. Tinker P. B. (1976). Translocation and transfer of nutrients in vesicular-arbuscular mycorrhizas i. the arbuscule and phosphorus transfer: a quantitative ultrastructural study. New Phytol. 77, 371–378. doi: 10.1111/j.1469-8137.1976.tb01526.x

[B11] Cui L. J. Liu X. Y. Lin J. Shi K. M. (2020). Effects of arbuscular mycorrhizal fungi on roots growth and endogenous hormones of *Phoebe zhennan* under salt stress. J. Nanjing For. Univ. (Nat. Sci.). 44, 119–124. doi: 10.3969/j.issn.1000-2006.201912030

[B12] Das D. Paries M. Hobecker K. Gigl M. Dawid C. Lam H. M.. (2022). Phosphate starvation response transcription factors enable arbuscular mycorrhiza symbiosis. Nat. Commun. 13, 477. doi: 10.1038/s41467-022-27976-8 35078978 PMC8789775

[B13] Duan J. Tian H. Drijber R. A. Gao Y. (2015). Systemic and local regulation of phosphate and nitrogen transporter genes by arbuscular mycorrhizal fungi in roots of winter wheat (*Triticum aestivum* L.). Plant Physiol. Bioch. 96, 199–208. doi: 10.1016/j.plaphy.2015.08.006 26298806

[B14] Duan J. F. Tian H. Gao Y. J. (2016). Effect of inoculating different arbuscular mycorrhizal fungi on the expression of nitrogen transporter genes in roots of wheat. Soil Fert. Sci. China., 130–136. doi: 10.11838/sfsc.20160224

[B15] Evangelina Q. A. Rincón-Enríquez E. Hernández-Cuevas G. Verónica L. Luis L. P. (2015). Influence of arbuscular mycorrhizal fungi and nitrogen concentrations on *Carica papaya* plant growth. Int. J. Agric. Biol. 17, 119−126.

[B16] Fonouni-Farde C. Miassod A. Laffont C. Morin H. Bendahmane A. Diet A.. (2019). Gibberellins negatively regulate the development of *Medicago truncatula* root system. Sci. Rep. 9, 2335. doi: 10.1038/s41598-019-38876-1 30787350 PMC6382856

[B17] Gao J. F. (2006). Experimental guidance for plant physiology (Beijing: Higher education press), 74–86.

[B18] Gao W. T. Zhang C. Y. Dong T. F. Xu X. (2019). Effects of arbuscular mycorrhizal fungi on the root growth of male and female *Populus cathayana* individuals grown under different sexual combination patterns. Chin. J. Plant Ecol. 43, 37–45. doi: 10.17521/cjpe.2018.0261

[B19] Gou J. Strauss S. H. Tsai C. J. Fang K. Chen Y. Jiang X.. (2010). Gibberellins regulate lateral root formation in *Populus* through interactions with auxin and other hormones. Plant Cell 22, 623–639. doi: 10.1105/tpc.109.073239 20354195 PMC2861444

[B20] Govindarajulu M. Pfeffer P. E. Jin H. Abubaker J. Douds D. D. Allen J. W.. (2005). Nitrogen transfer in the arbuscular mycorrhizal symbiosis. Nature 435, 819–823. doi: 10.1038/nature03610 15944705

[B21] Harris J. M. (2015). Abscisic acid: hidden architect of root system structure. Plants 4, 548–572. doi: 10.3390/plants4030548 27135341 PMC4844405

[B22] Hawkins H. J. Johansen A. George E. (2000). Uptake and transport of organic and inorganic nitrogen by arbuscular mycorrhizal fungi. Plant Soil 226, 275–285. doi: 10.1023/A:1026500810385

[B23] Hermans C. Hammond J. P. White P. J. Verbruggen N. (2006). How do plants respond to nutrient shortage by biomass allocation? Trends Plant Sci. 11, 610–617. doi: 10.1016/j.tplants.2006.10.007 17092760

[B24] Hildebrandt U. Schmelzer E. Bothe H. (2002). Expression of nitrate transporter genes in tomato colonized by an arbuscular mycorrhizal fungus. Physiol. Plantarum 115, 125–136. doi: 10.1034/j.1399-3054.2002.1150115.x 12010476

[B25] Huang W. T. Zheng Z. C. Hua D. Chen X. F. Zhang J. Chen H. H.. (2022). Adaptive responses of carbon and nitrogen metabolisms to nitrogen-deficiency in *Citrus sinensis* seedlings. BMC Plant Biol. 22, 370. doi: 10.1186/s12870-022-03759-7 35879653 PMC9316421

[B26] Huang X. H. Zhu F. Hu F. J. Liang H. Z. Wang R. J. Zou Z. G. (2018). Effects of Pb stress on chlorophyll fluorescence of *Schima superba* and *Koelreuteria paniculata* seedling based on lake-model. Acta Ecol. Sin. 38, 1284–1292. doi: 10.5846/stxb201701160123

[B27] Jian S. Zhu T. Wang J. Yan D. (2022). The current and future potential geographical distribution and evolution process of *Catalpa bungei* in China. Forests 13, 96. doi: 10.3390/f13010096

[B28] Kaldorf M. Schmelzer E. Bothe H. (1998). Expression of maize and fungal nitrate reductase genes in arbuscular mycorrhiza. Mol. Plant-Microbe In. 11, 439–448. doi: 10.1094/MPMI.1998.11.6.439 9612942

[B29] Kang F. Yang B. Yang W. Wang L. Guo J.. (2020). Arbuscular mycorrhizal fungi alleviate the negative effect of nitrogen deposition on ecosystem functions in meadow grassland. Land Degrad. Dev. 31, 748–759. doi: 10.1002/ldr.3491

[B30] Khan Y. Yang X. Zhang X. Yaseen T. Shi L. Zhang T. (2021). Arbuscular mycorrhizal fungi promote plant growth of *Leymus chinensis* (Trin.) tzvelev by increasing the metabolomics activity under nitrogen addition. Grassl. Sci. 67, 128–138. doi: 10.1111/grs.12299

[B31] Kong C. B. Pang Z. Q. Zhang C. F. Liu Q. Hu C. H. Xiao Y. J.. (2022). Effects of arbuscular mycorrhizal fungi on sugarcane growth and nutrient related gene co-expression network under different fertilization levels. Acta Agron. Sin. 48, 860–872. doi: 10.3724/SP.J.1006.2022.14052

[B32] Li Q. S. Xie Y. C. Rahman M. M. Hashem A. Allah E. F. Wu Q. S. (2022). Arbuscular mycorrhizal fungi and endophytic fungi activate leaf antioxidant defense system of lane late navel orange. J. Fungi 8, 282. doi: 10.3390/jof8030282 PMC8954850 35330284

[B33] Livak K. J. Schmittgen T. D. (2001). Analysis of relative gene expression data using real-time quantitative PCR and the 2^–ΔΔCT^ method. Methods 25, 402–408. doi: 10.1006/meth.2001.1262 11846609

[B34] Lu Y. Ma Q. Chen C. Xu X. Zhang D. (2021). Effects of arbuscular mycorrhizal fungi on the nitrogen distribution in endangered *Torreya jackii* under nitrogen limitation. Planta 254, 53. doi: 10.1007/s00425-021-03704-2 34402996

[B35] Luo J. Li H. Liu T. Polle A. Peng C. Luo Z. B. (2013). Nitrogen metabolism of two contrasting poplar species during acclimation to limiting nitrogen availability. J. Exp. Bot. 64, 4207–4224. doi: 10.1093/jxb/ert234 23963674 PMC3808312

[B36] Lv F. Wang P. Zhang E. Ma L. Gao L. Yang R.. (2021). Efficient transformation of *Catalpa bungei* shows *Crystal* genes conferring resistance to the shoot borer *Omphisa plagialis* . Front. Plant Sci. 12. doi: 10.3389/fpls.2021.777411 PMC8739885 35003162

[B37] Ma S. Zhu L. Wang J. Liu X. Jia Z. Li C.. (2022). Arbuscular mycorrhizal fungi promote *Gleditsia sinensis* lam. root growth under salt stress by regulating nutrient uptake and physiology. Forests 13, 688. doi: 10.3390/f13050688

[B38] McGonigle T. P. Miller M. H. Evans D. G. Fairchild G. L. Swan J. A. (1990). A new method which gives an objective measure of colonization of roots by vesicular-arbuscular mycorrhizal fungi. New Phytol. 115, 495–501. doi: 10.1111/j.1469-8137.1990.tb00476.x 33874272

[B39] Meng Q. S. Qin Q. Q. Liu Y. H. (2022). Effects of nitrogen application on growth, development and physiological characteristics of *Taxus cuspidata* seedlings. Chin. J. Ecol. 41, 2325–2334. doi: 10.13292/j.1000-4890.202211.007

[B40] Ouahmane L. Hafidi M. Thioulouse J. Ducousso M. Kisa M. Prin Y.. (2007). Improvement of *Cupressus atlantica* gaussen growth by inoculation with native arbuscular mycorrhizal fungi. J. Appl. Microbiol. 103, 683–690. doi: 10.1111/j.1365-2672.2007.03296.x 17714402

[B41] Outamamat E. Dounas H. Aziz F. Barguaz A. Duponnois R. Ouahmane L. (2022). The first use of morphologically isolated arbuscular mycorrhizal fungi single-species from Moroccan ecosystems to improve growth, nutrients uptake and photosynthesis in *Ceratonia siliqua* seedlings under nursery conditions. Saudi J. Biol. Sci. 29, 2121–2130. doi: 10.1016/j.sjbs.2021.11.049 35531245 PMC9072874

[B42] Pan X. Welti R. Wang X. (2010). Quantitative analysis of major plant hormones in crude plant extracts by high-performance liquid chromatography-mass spectrometry. Nat. Protoc. 5, 986–992. doi: 10.1038/nprot.2010.37 20448544

[B43] Pérez-Tienda J. Corrêa A. Azcón-Aguilar C. Ferrol N. (2014). Transcriptional regulation of host ${\text{NH}}_{4}^{+}$ transporters and GS/GOGAT pathway in arbuscular mycorrhizal rice roots. Plant Physiol. Bioch. 75, 1–8. doi: 10.1016/j.plaphy.2013.11.029 24361504

[B44] Phillips J. M. Hayman D. S. (1970). Improved procedures for clearing roots and staining parasitic and vesicular-arbuscular mycorrhizal fungi for rapid assessment of infection. Trans. Br. Mycol. Soc 55, 158–161. doi: 10.1016/S0007-1536(70)80110-3

[B45] Qin F. Yu S. (2019). Arbuscular mycorrhizal fungi protect native woody species from novel weapons. Plant Soil 440, 39–52. doi: 10.1007/s11104-019-04063-4

[B46] Qin J. Yue X. Fang S. Qian M. Zhou S. Shang X.. (2021). Responses of nitrogen metabolism, photosynthetic parameter and growth to nitrogen fertilization in *Cyclocarya paliurus* . For. Ecol. Manage. 502, 119715. doi: 10.1016/j.foreco.2021.119715

[B47] Ren W. Guo Y. Han X. Sun Y. Li Q. Wu B.. (2022). Indigenous microorganisms offset arbuscular mycorrhizal fungi-induced plant growth and nutrient acquisition through negatively modulating the genes of phosphorus transport and nitrogen assimilation. Front. Plant Sci. 13. doi: 10.3389/fpls.2022.880181 PMC9125159 35615141

[B48] Riaz M. Kamran M. Fang Y. Wang Q. Cao H. Yang G.. (2021). Arbuscular mycorrhizal fungi-induced mitigation of heavy metal phytotoxicity in metal contaminated soils: A critical review. J. Hazard. Mater. 402, 123919. doi: 10.1016/j.jhazmat.2020.123919 33254825

[B49] Rooney D. C. Prosser J. I. Bending G. D. Baggs E. M. Killham K. Hodge A. (2011). Effect of arbuscular mycorrhizal colonisation on the growth and phosphorus nutrition of *Populus euramericana* cv ghoy. Biomass Bioenergy 35, 4605–4612. doi: 10.1016/j.biombioe.2011.08.015

[B50] Roussis I. Beslemes D. Kosma C. Triantafyllidis V. Zotos A. Tigka E.. (2022). The influence of arbuscular mycorrhizal fungus *Rhizophagus irregularis* on the growth and quality of processing tomato (*Lycopersicon esculentum* mill.) seedlings. Sustainability 14, 9001. doi: 10.3390/su14159001

[B51] Saia S. Jansa J. (2022). Editorial: arbuscular mycorrhizal fungi: the bridge between plants, soils, and humans. Front. Plant Sci. 13. doi: 10.3389/fpls.2022.875958 PMC9014169 35444670

[B52] Salifu K. F. Timmer V. R. (2003). Optimizing nitrogen loading of *Picea mariana* seedlings during nursery culture. Can. J. For. Res. 33, 1287–1294. doi: 10.1139/x03-057

[B53] Savolainen T. Kytöviita M. M. (2022). Mycorrhizal symbiosis changes host nitrogen source use. Plant Soil 471, 643–654. doi: 10.1007/s11104-021-05257-5

[B54] Seepaul R. George S. Wright D. L. (2016). Comparative response of *Brassica carinata* and *B. napus* vegetative growth, development and photosynthesis to nitrogen nutrition. Ind. Crop Prod. 94, 872–883. doi: 10.1016/j.indcrop.2016.09.054

[B55] Shan L. W. Zhang Q. Zhu R. F. Kong X. L. Chen J. S. (2020). Effects of AMF on growth and photosynthetic physiological characteristics of *Leymus chinensis* and *Medicago sativa* with and without nitrogen and phosphorus application. Acta Pratac. Sin. 29, 46–57. doi: 10.11686/cyxb2019459

[B56] Singh S. Pandey A. Kumar B. Palni L. M. S. (2010). Enhancement in growth and quality parameters of tea [*Camellia sinensis* (L.) o. kuntze] through inoculation with arbuscular mycorrhizal fungi in an acid soil. Biol. Fertil. Soils 46, 427–433. doi: 10.1007/s00374-010-0448-x

[B57] Smith S. E. Smith F. A. (2011). Roles of arbuscular mycorrhizas in plant nutrition and growth: new paradigms from cellular to ecosystem scales. Annu. Rev. Plant Biol. 62, 227–250. doi: 10.1146/annurev-arplant-042110-103846 21391813

[B58] Song X. Wan F. Chang X. Zhang J. Sun M. Liu Y. (2019). Effects of nutrient deficiency on root morphology and nutrient allocation in *Pistacia chinensis* bunge seedlings. Forests 10, 1035. doi: 10.3390/f10111035

[B59] Sun T. Zhang J. Zhang Q. Li X. Li M. Yang Y.. (2021). Integrative physiological, transcriptome, and metabolome analysis reveals the effects of nitrogen sufficiency and deficiency conditions in apple leaves and roots. Environ. Exp. Bot. 192, 104633. doi: 10.1016/j.envexpbot.2021.104633

[B60] Tanaka Y. Yano K. (2005). Nitrogen delivery to maize *via* mycorrhizal hyphae depends on the form of n supplied. Plant Cell Environ. 28, 1247–1254. doi: 10.1111/j.1365-3040.2005.01360.x

[B61] Timmer V. R. (1997). Exponential nutrient loading: a new fertilization technique to improve seedling performance on competitive sites. New Forest. 13, 279–299. doi: 10.1023/A:1006502830067

[B62] Tomazelli D. Costa M. D. Primieri S. Rech T. D. Santos J. C. P. Klauberg-Filho O. (2022). Inoculation of arbuscular mycorrhizal fungi improves growth and photosynthesis of *Ilex paraguariensis* (St. hil) seedlings. Braz. Arch. Biol. Techn. 65, e22210333. doi: 10.1590/1678-4324-2022210333

[B63] Turjaman M. Tamai Y. Santoso E. Osaki M. Tawaraya K. (2006). Arbuscular mycorrhizal fungi increased early growth of two nontimber forest product species *Dyera polyphylla* and *Aquilaria filaria* under greenhouse conditions. Mycorrhiza 16, 459–464. doi: 10.1007/s00572-006-0059-4 16758200

[B64] Ubeda-Tomás S. Federici F. Casimiro I. Beemster G. T. Bhalerao R. Swarup R.. (2009). Gibberellin signaling in the endodermis controls *Arabidopsis* root meristem size. Curr. Biol. 19, 1194–1199. doi: 10.1016/j.cub.2009.06.023 19576770

[B65] Veresoglou S. D. Menexes G. Rillig M. C. (2012). Do arbuscular mycorrhizal fungi affect the allometric partition of host plant biomass to shoots and roots? a meta-analysis of studies from 1990 to 2010. Mycorrhiza 22, 227–235. doi: 10.1007/s00572-011-0398-7 21710352

[B70] Wang Y. X. Li Q. Shen Y. K. Yang Q. Zhang J. B. Wang Y. H.. (2021b). Effects of nitrogen deposition on arbuscular myorrhizal fungal colonization and glomalin-related soil protein of Chinese fir. Acta Ecol. Sin. 41, 194–201. doi: 10.5846/stxb201912112692

[B66] Wang J. Qin X. Xu S. Zhao M. Shu P. Xu F.. (2021a). Nitrogen availability affects stem development and response to differential root-zone drought stress in *Catalpa bungei* . Environ. Exp. Bot. 186, 104429. doi: 10.1016/j.envexpbot.2021.104429

[B68] Wang R. Shi L. Wang Y. (2022). Physical and mechanical properties of *Catalpa bungei* clones and estimation of the properties by near-infrared spectroscopy. J. Renew. Mater. 10, 3285–3302. doi: 10.32604/jrm.2022.020546

[B69] Wang Y. M. Wan F. X. Li R. R. Hu F. Zhang H. Ju C. H.. (2018). Effects of exponential fertilization on growth and nutrient accumulation of *Carya illinoensis* seedlings. J. Northeast For. Univ. 46, 21–25. doi: 10.13759/j.cnki.dlxb.2018.09.005

[B67] Wang J. Zhong H. Zhu L. Yuan Y. Xu L. Wang G. G.. (2019). Arbuscular mycorrhizal fungi effectively enhances the growth of *Gleditsia sinensis* lam. seedlings under greenhouse conditions. Forests 10, 567. doi: 10.3390/f10070567

[B71] Weng W. Yan J. Zhou M. Yao X. Gao A. Ma C.. (2022). Roles of arbuscular mycorrhizal fungi as a biocontrol agent in the control of plant diseases. Microorganisms 10, 1266. doi: 10.3390/microorganisms10071266 35888985 PMC9317293

[B74] Wu J. W. He Q. Li J. Y. Wang J. H. Su Y. Wang L. P.. (2015). Dynamic changes of foliage growth of *Catalpa bungei* clones under different nitrogen exponential fertilizations. J. Beijing For. Univ. 37, 19–28. doi: 10.13332/j.1000-1522.20140437

[B72] Wu F. Zhang H. Fang F. Liu H. Tang M. (2017a). Arbuscular mycorrhizal fungi alter nitrogen allocation in the leaves of *Populus × canadensis* ‘Neva’. Plant Soil 421, 477–491. doi: 10.1007/s11104-017-3461-0

[B73] Wu F. Zhang H. Fang F. Wu N. Zhang Y. Tang M. (2017b). Effects of nitrogen and exogenous *Rhizophagus irregularis* on the nutrient status, photosynthesis and leaf anatomy of *Populus × canadensis* ‘Neva’. J. Plant Growth Regul. 36, 824–835. doi: 10.1007/s00344-017-9686-6

[B75] Xiao D. Wang X. J. Zhang K. Kang F. F. He N. P. Hou J. H. (2015). Effects of simulated nitrogen deposition on growth of *Acer mono* seedlings. J. Beijing For. Univ. 37, 50–57. doi: 10.13332/j.1000-1522.20150079

[B77] Yang Y. Tang M. Sulpice R. Chen H. Tian S. Ban Y. (2014). Arbuscular mycorrhizal fungi alter fractal dimension characteristics of *Robinia pseudoacacia* L. seedlings through regulating plant growth, leaf water status, photosynthesis, and nutrient concentration under drought stress. J. Plant Growth Regul. 33, 612–625. doi: 10.1007/s00344-013-9410-0

[B76] Yang R. Zhang H. Hu L. Fan Z. (2021). Effects of AMF inoculation and nitrogen application on nitrogen mineralization of coastal saline soil. J. Nanjing For. Univ. (Nat. Sci.) 45, 145–152. doi: 10.12302/j.issn.1000-2006.202003087

[B78] Zhang H. Liu Z. Chen H. Tang M. (2016). Symbiosis of arbuscular mycorrhizal fungi and *Robinia pseudoacacia* L. improves root tensile strength and soil aggregate stability. PloS One 11, e0153378. doi: 10.1371/journal.pone.0153378 27064570 PMC4827865

[B79] Zhang M. Lu N. Zhu T. Yang G. Qu G. Shi C.. (2021). A bivariate mapping model identifies major covariation QTLs for biomass allocation between leaf and stem growth of *Catalpa bungei* . Front. Genet. 12. doi: 10.3389/fgene.2021.758209 PMC8637733 34868235

[B80] Zhao Q. H. Sun L. T. Wang Y. Ding Z. T. Li M. (2014). Effects of arbuscular mycorrhizal fungi and nitrogen regimes on plant growth, nutrient uptake and tea quality in *Camellia sinensis* (L.) o. kuntze. Plant Physiol. J. (In Chinese) 50, 164–170. doi: 10.13592/j.cnki.ppj.2014.02.007

[B81] Zheng H. Zhang X. Ma W. Song J. Rahman S. U. Wang J.. (2017). Morphological and physiological responses to cyclic drought in two contrasting genotypes of *Catalpa bungei* . Environ. Exp. Bot. 138, 77–87. doi: 10.1016/j.envexpbot.2017.02.016

[B500] Zhu X. Q. Wang C. Y. Chen H. Tang M. (2014). Effects of arbuscular mycorrhizal fungi on photosynthesis, carbon content, and calorific value of black locust seedlings. Photosynthetica 52, 247–252. doi: 10.1007/s11099-014-0031-z

