# Supplementary material for: Effects of arbuscular mycorrhizal fungus inoculation on the growth and nitrogen metabolism of Catalpa bungei C.A.Mey. under different nitrogen levels
Source: Front Plant Sci. 2023 Feb 23;14:1138184. doi: 10.3389/fpls.2023.1138184 (PMC9996104; doi:10.3389/fpls.2023.1138184)
Supplement: Supplementary file 1 [file DataSheet_1.docx]

***Supplementary Material***

**Effects of arbuscular mycorrhizal fungus inoculation on the growth and nitrogen metabolism of *Catalpa bungei* C.A.Mey. under different nitrogen levels**

**Wei Chen^1^, Xueli Mou^1^, Panpan Meng^1^, Juan Chen^1^, Xiaan Tang^1^, Guihua Meng^1^, Kexu Xin^1^, Yi Zhang^1^, Chunyan Wang^1^***

*** Correspondence:** chunyan@nwsuaf.edu.cn

**Supplementary Data**

**
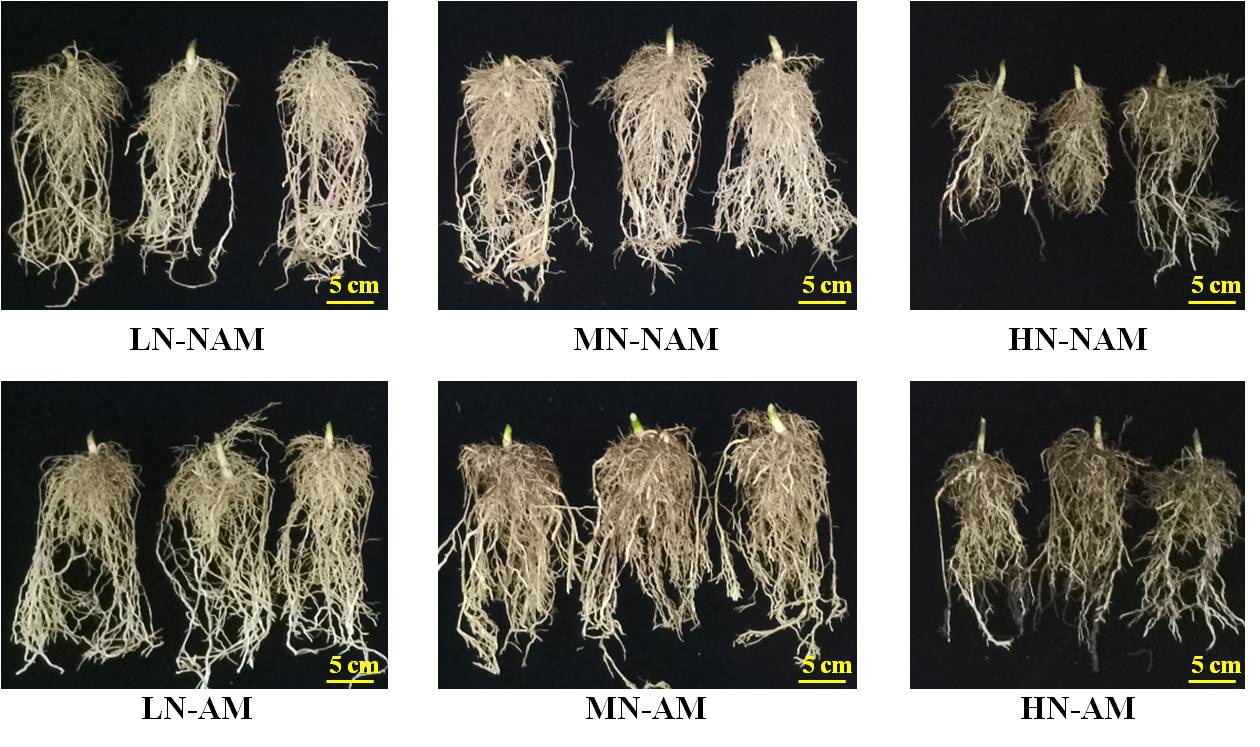
**

**Fig. S1.** Effect of the arbuscular mycorrhizal fungus (AMF) *Rhizophagus intraradices* on the root morphology of *Catalpa* *bungei* seedlings under different nitrogen (N) levels. LN, low N level (0.25 mM); MN, moderate N level (10 mM); HN, high N level (45 mM); NAM, non-AMF-inoculated; AM, AMF-inoculated.

**
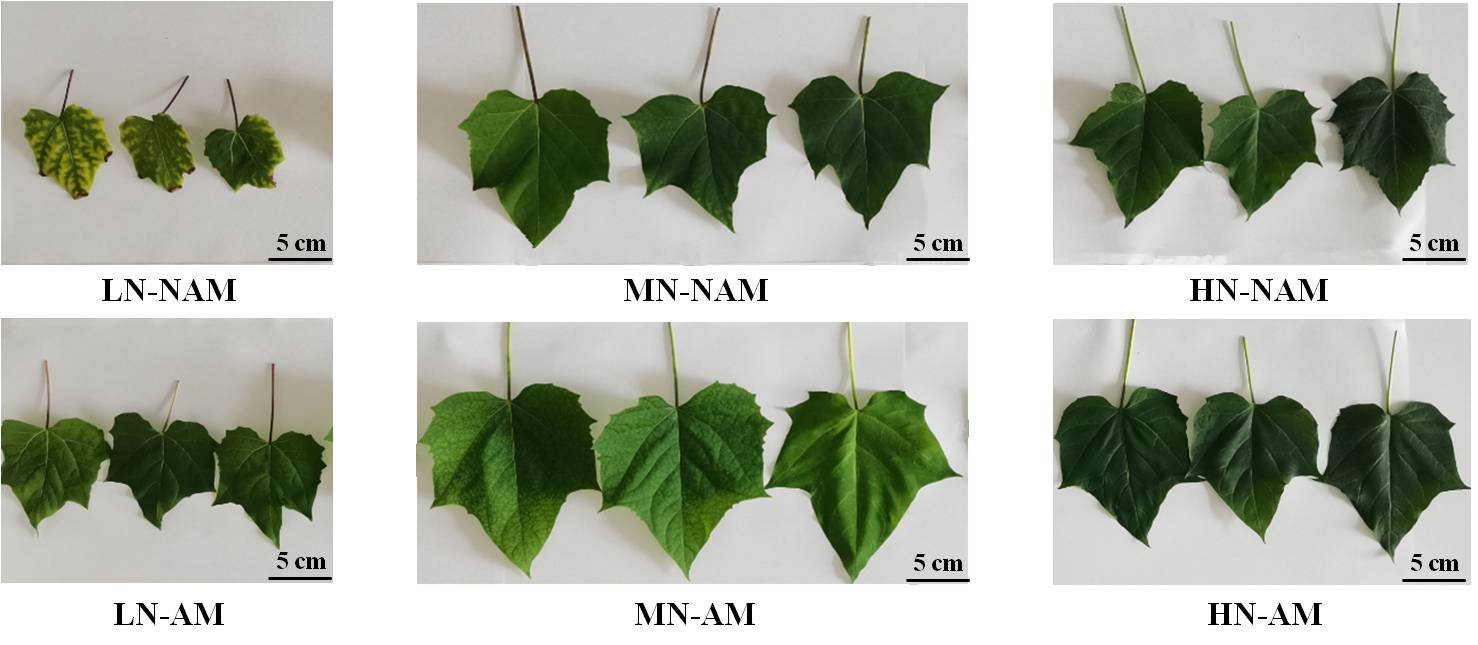
**

**Fig. S2.** Effect of the arbuscular mycorrhizal fungus (AMF) *Rhizophagus intraradices* on the leaf morphology of *Catalpa* *bungei* under different nitrogen (N) levels. LN, low N level (0.25 mM); MN, moderate N level (10 mM); HN, high N level (45 mM); NAM, non-AMF-inoculated; AM, AMF-inoculated.


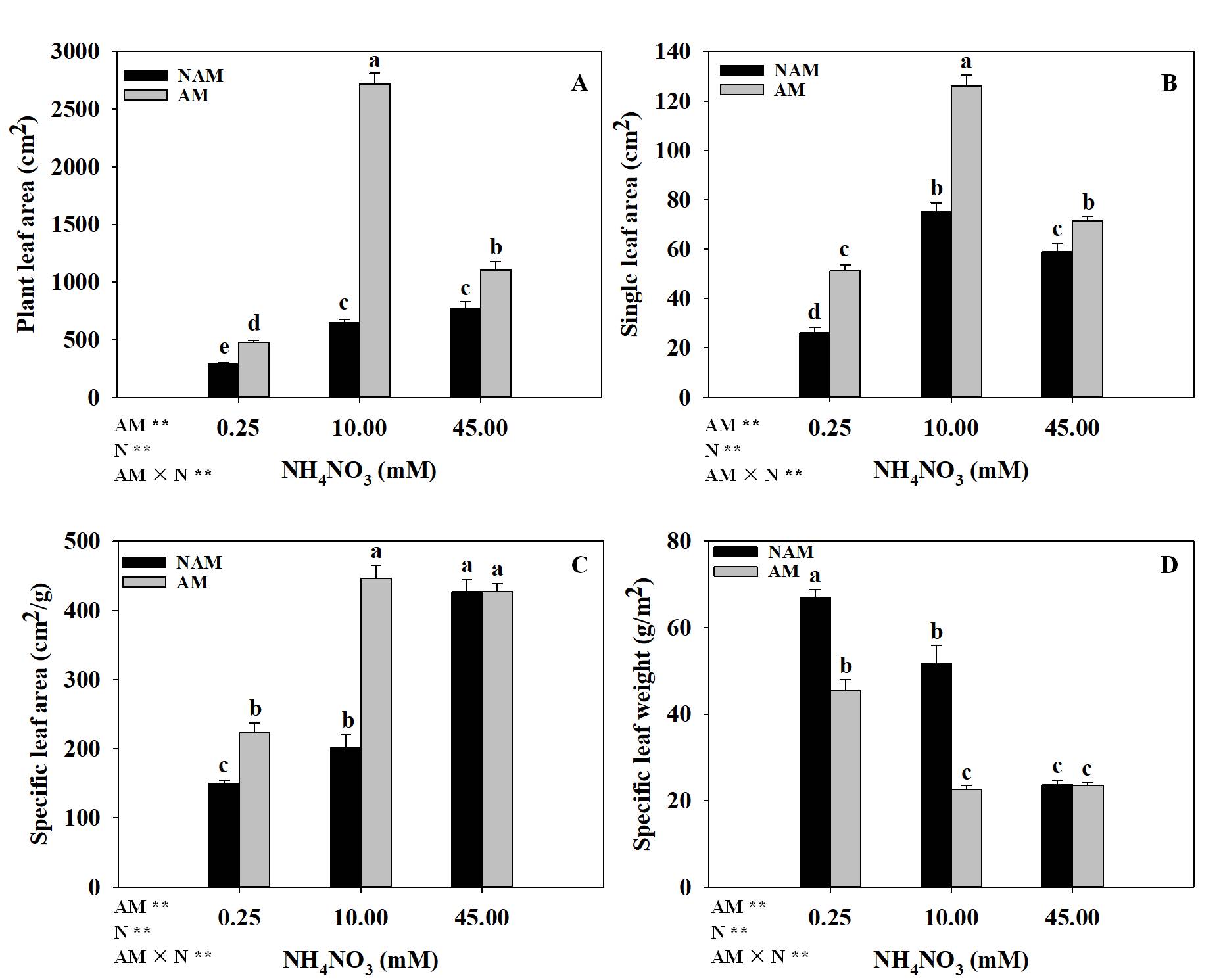


**Fig. S3.** Effect of the arbuscular mycorrhizal fungus (AMF) *Rhizophagus intraradices* on **(A)** plant leaf area, **(B)** single leaf area, **(C)** specific leaf area (SLA) and **(D)** specific leaf weight (SLW) of *Catalpa* *bungei* seedlings under different nitrogen (N) levels. NAM, non-AMF-inoculated; AM, AMF-inoculated. Different lowercase letters above the bars indicate significant differences (*p* < 0.05) among treatments. Values are means ± SE (*n* = 6). Two-way ANOVA output: ns, not significant; * *p* < 0.05; ** *p* < 0.01.

**Table S1.** Blastx of *Catalpa bungei* CDSs responsible for nitrogen (N) metabolism and primers used for qRT-PCR

| Gene Classes | Gene Name | Closest Reference Sequence  via blastx in NCBI | Query Cover | Identity | Forward primer  (From left to right 5'-3') | Reverse primer  (From left to right 5'-3') |
| --- | --- | --- | --- | --- | --- | --- |
| Nitrogen metabolism and transport | NR | [*Sesamum indicum*] XM_011099324.2 | 100% | 83.68% | GTGGACGACTCAACGACCAT | CCAGCACCAATACTTCCCGT |
|  | Fd-NiR | [*Sesamum indicum*] XM_011089958.2 | 100% | 84.12% | TGGCGTTGTTTTGCCTGATG | AGCATTCCACTTCCTTGGCA |
|  | NADH-GOGAT1 | [*Sesamum indicum*] XM_011077706.2 | 98% | 89.42% | CTTCTTGGTGCGGAGGAGTT | GAACTGGATCTTGGGTGGCA |
|  | Fd-GOGAT | [*Sesamum indicum*] XM_011077112.2 | 80% | 90.43% | AATGGTCCCGTTTTGGACGA | CAAGCAAACGACTGACCAGC |
|  | GDHA | [*Sesamum indicum*] XM_011086464.2 | 100% | 88.03% | TCTCGGTGGTTCATTGGGTC | CGACCTTGCCACCTCTTTCA |
|  | GDHB | [*Sesamum indicum*] XM_020693092.1 | 100% | 91.83% | GGTTCAGCACGACAATGCTC | GCTCAAGTCTGATGGGTTGC |
|  | NADP-GDH | [*Sesamum indicum*] XM_020692550.1 | 99% | 91.85% | ACGACACTCCGTTTTCCCAT | TCGCGGCATACAGAACAGAA |
|  | GS | [*Avicennia marina*] AF338444.1 | 98% | 86.47% | ATTTTCAGCCACCCCGATGT | AGCCTTGTCAGCTCCAACTC |
|  | GS1 | [*Sesamum indicum*] XM_011084818.2 | 99% | 89.32% | GGTGGCGCGTTACATTCTTG | GTTCGCCACACCCCATAAGA |
|  | NRT2.4 | [*Sesamum indicum*] XM_011090946.2 | 100% | 82.33% | CGGGAACTTTGGCTCTGGAT | TCATCCACACCTTTCGACGG |
|  | NRT2.5 | [*Sesamum indicum*] XM_011072822.2 | 98% | 83.95% | CGCTTCCTCCGCTCTAACTT | AGTCGCACCGATTTGATGGA |
|  | NRT2.7 | [*Juglans microcarpa* × *Juglans regia*] XM_041150633.1 | 94% | 72.02% | CGACGAAATGGGACGGAGAT | CACGACGCCAAATGTAAGGC |

**Table S2.** PCA of growth and physiological characteristics of *Catalpa bungei* seedlings under nitrogen (N) application and mycorrhizal inoculation.

| Variables | PC1 | PC2 | PC3 | PC4 | PC5 | PC6 |
| --- | --- | --- | --- | --- | --- | --- |
| Plant height | 0.956 | 0.034 | -0.000 | 0.216 | -0.082 | 0.136 |
| Basal diameter | 0.603 | -0.009 | -0.537 | 0.368 | 0.068 | 0.318 |
| Total biomass | 0.832 | 0.225 | -0.380 | 0.143 | -0.102 | 0.234 |
| Plant leaf area | 0.912 | -0.006 | -0.229 | -0.192 | -0.212 | 0.161 |
| Single leaf area | 0.972 | -0.083 | -0.027 | -0.012 | -0.066 | 0.053 |
| Chlorophyll a | 0.826 | 0.244 | 0.266 | 0.297 | -0.242 | -0.307 |
| Chlorophyll b | 0.866 | 0.212 | 0.223 | 0.21 | -0.327 | -0.283 |
| Chlorophyll a+b | 0.839 | 0.236 | 0.255 | 0.276 | -0.265 | -0.301 |
| Carotenoids | 0.826 | 0.337 | 0.134 | 0.393 | -0.157 | -0.206 |
| Pn | 0.867 | -0.113 | -0.219 | -0.042 | 0.252 | -0.267 |
| Gs | 0.383 | 0.645 | -0.634 | -0.110 | 0.188 | -0.141 |
| Ci | -0.060 | 0.737 | -0.569 | 0.030 | 0.150 | 0.109 |
| Tr | 0.015 | 0.078 | -0.846 | 0.173 | 0.228 | -0.492 |
| WUE | 0.696 | -0.208 | 0.525 | -0.157 | -0.047 | 0.291 |
| Fv/Fm | 0.828 | -0.406 | 0.131 | -0.039 | 0.130 | 0.025 |
| Fv/Fo | 0.854 | -0.400 | 0.083 | -0.040 | 0.134 | 0.017 |
| ΦPSⅡ | 0.772 | -0.517 | -0.111 | -0.166 | 0.234 | 0.115 |
| rETR | 0.772 | -0.517 | -0.110 | -0.167 | 0.235 | 0.116 |
| qP | 0.751 | -0.399 | -0.150 | -0.438 | 0.097 | 0.064 |
| NPQ | -0.111 | 0.493 | -0.200 | -0.607 | -0.322 | -0.001 |
| GA_3_ | 0.655 | 0.684 | -0.172 | 0.074 | -0.010 | 0.353 |
| IAA | 0.169 | 0.827 | 0.163 | -0.492 | 0.012 | 0.046 |
| CTK | 0.299 | 0.705 | 0.432 | -0.137 | 0.399 | 0.044 |
| ABA | -0.059 | 0.687 | 0.530 | -0.163 | 0.305 | 0.216 |
| NR | 0.234 | 0.408 | 0.230 | 0.646 | 0.354 | 0.148 |
| NiR | 0.160 | 0.262 | -0.077 | -0.636 | -0.035 | -0.279 |
| GOGAT | 0.537 | -0.160 | 0.066 | -0.488 | 0.379 | -0.332 |
| GDH | 0.370 | 0.156 | 0.682 | -0.188 | 0.144 | -0.229 |
| GS | 0.400 | 0.029 | -0.196 | -0.567 | -0.406 | 0.183 |
| Eigenvalue | 11.242 | 4.572 | 3.328 | 2.733 | 1.340 | 1.314 |
| Proportion of variation (%) | 38.76 | 15.76 | 11.48 | 9.43 | 4.62 | 4.53 |
| Cumulative proportion (%) | 38.76 | 54.52 | 66.00 | 75.43 | 80.05 | 84.58 |

Pn, net photosynthetic rate; Gs, stomatal conductance; Tr, transpiration rate; Ci, intercellular CO_2_ concentration; WUE, water use efficiency; Fv/Fm, maximum photochemical efficiency of PSII; Fv/Fo, potential photochemical efficiency of PSII; ФPSII, actual photochemical efficiency of PSII; rETR, relative electron transport rate through PSII; qP, photochemical quenching; NPQ, non-photochemical quenching; GA_3_, gibberellin; IAA, indole-3-acetic acid; CTK, cytokinin; ABA, abscisic acid; NR, nitrate reductase; NiR, nitrite reductase; GOGAT, glutamate synthetase; GDH, glutamate dehydrogenase; GS, glutamine synthetase.

**Table S3.** Pearson correlation coefficient between the percentage of mycorrhizal colonization and other parameters.

| Variable | Mycorrhizal colonization | | | | Variable | Mycorrhizal colonization | | | |
| --- | --- | --- | --- | --- | --- | --- | --- | --- | --- |
|  | Hyphae | Arbuscules | Vesicles | Total |  | Hyphae | Arbuscules | Vesicles | Total |
| Plant height | 0.708** | 0.923** | 0.824** | 0.904** | Stem P | -0.426 | -0.061 | -0.345 | -0.266 |
| Basal diameter | 0.759** | 0.935** | 0.824** | 0.883** | Root P | -0.434 | -0.299 | -0.015 | -0.273 |
| Leaf number | 0.659** | 0.805** | 0.680** | 0.839** | Leaf K | -0.331 | -0.633** | -0.317 | -0.625** |
| Leaf biomass | 0.796** | 0.943** | 0.795** | 0.892** | Stem K | -0.463 | -0.789** | -0.496* | -0.685** |
| Stem biomass | 0.702** | 0.947** | 0.834** | 0.907** | Root K | -0.897** | -0.419 | -0.423 | -0.520* |
| Root biomass | 0.171 | 0.778** | 0.624** | 0.664** | Leaf Ca | 0.359 | -0.255 | -0.213 | -0.085 |
| Total biomass | 0.684** | 0.963** | 0.809** | 0.895** | Stem Ca | 0.834** | 0.517* | 0.355 | 0.562* |
| LMR | 0.761** | 0.166 | 0.162 | 0.226 | Root Ca | 0.691** | 0.932** | 0.808** | 0.906** |
| SMR | 0.366 | 0.426 | 0.400 | 0.498* | Leaf Mg | 0.742** | 0.440 | 0.584* | 0.568* |
| RMR | -0.799** | -0.232 | -0.223 | -0.301 | Stem Mg | 0.395 | 0.639** | 0.757** | 0.708** |
| Root/shoot ratio | -0.792** | -0.254 | -0.233 | -0.317 | Root Mg | 0.282 | 0.820** | 0.709** | 0.726** |
| Plant leaf area | 0.881** | 0.905** | 0.771** | 0.882** | Root length | 0.411 | 0.896** | 0.699** | 0.819** |
| Single leaf area | 0.827** | 0.897** | 0.735** | 0.885** | Root projected area | 0.398 | 0.893** | 0.692** | 0.801** |
| SLA | 0.889** | 0.434 | 0.403 | 0.529* | Root surface area | 0.398 | 0.893** | 0.692** | 0.801** |
| SLW | -0.881** | -0.412 | -0.375 | -0.520* | Root volume | 0.356 | 0.848** | 0.648** | 0.744** |
| Chlorophyll a | 0.250 | 0.657** | 0.423 | 0.628** | Root tips | 0.598** | 0.817** | 0.613** | 0.806** |
| Chlorophyll b | 0.406 | 0.734** | 0.510* | 0.717** | Root forks | 0.469* | 0.828** | 0.678** | 0.797** |
| Chlorophyll a+b | 0.293 | 0.680** | 0.448 | 0.654** | Fine root length | 0.386 | 0.845** | 0.690** | 0.801** |
| Chlorophyll a/b | -0.903** | -0.636** | -0.621** | -0.681** | Fine root surface area | 0.427 | 0.899** | 0.722** | 0.830** |
| Carotenoids | 0.229 | 0.717** | 0.489* | 0.663** | Length per unit volume | 0.411 | 0.896** | 0.699** | 0.819** |
| Pn | 0.811** | 0.615** | 0.536* | 0.663** | Average root diameter | -0.247 | -0.173 | -0.228 | -0.302 |
| Gs | 0.193 | 0.556* | 0.507* | 0.472* | Root activity | 0.255 | 0.767** | 0.700** | 0.724** |
| Ci | -0.277 | 0.342 | 0.226 | 0.163 | Root IAA | -0.645** | -0.027 | -0.001 | -0.154 |
| Tr | 0.442 | 0.355 | 0.347 | 0.432 | Root CTK | -0.769** | -0.204 | -0.200 | -0.354 |
| WUE | 0.528* | 0.414 | 0.252 | 0.381 | Root GA_3_ | 0.154 | 0.735** | 0.647** | 0.600** |
| Fv/Fm | 0.822** | 0.515* | 0.487* | 0.621** | Root ABA | -0.789** | -0.341 | -0.315 | -0.464 |
| Fv/Fo | 0.819** | 0.531* | 0.503* | 0.630** | IAA/ABA | 0.392 | 0.662** | 0.595** | 0.651** |
| ΦPSⅡ | 0.883** | 0.514* | 0.416 | 0.618** | CTK/ABA | 0.489* | 0.455 | 0.338 | 0.481* |
| rETR | 0.883** | 0.513* | 0.414 | 0.616** | GA_3_/ABA | 0.515* | 0.912** | 0.789** | 0.829** |
| qP | 0.869** | 0.504* | 0.349 | 0.530* | Root NR | -0.403 | 0.216 | 0.133 | 0.177 |
| NPQ | -0.246 | 0.128 | -0.021 | -0.095 | Root NiR | 0.106 | -0.201 | -0.290 | -0.154 |
| Leaf N | 0.503* | -0.246 | -0.161 | -0.092 | Root GS | 0.533* | 0.447 | 0.373 | 0.430 |
| Stem N | 0.391 | -0.359 | -0.265 | -0.184 | Root GOGAT | 0.422 | -0.071 | -0.008 | -0.044 |
| Root N | 0.435 | -0.315 | -0.239 | -0.167 | Root GDH | -0.247 | -0.366 | -0.287 | -0.403 |
| Leaf P | 0.283 | -0.353 | -0.269 | -0.241 |  |  |  |  |  |

LMR, Leaf mass ratio; SMR, Stem mass ratio; RMR, Root mass ratio; SLA, specific leaf area; SLW, specific leaf weight; Pn, net photosynthetic rate; Gs, stomatal conductance; Ci, intercellular CO_2_ concentration; Tr, transpiration rate; WUE, Water use efficiency; Fv/Fm, maximum photochemical efficiency of PSII; Fv/Fo, potential photochemical efficiency of PSII; ФPSII, actual photochemical efficiency of PSII; rETR, relative electron transport rate through PSII; NPQ, non-photochemical quenching; qP, photochemical quenching; IAA, indole-3-acetic acid; CTK, cytokinin; GA_3_, gibberellin; ABA, abscisic acid; NR, nitrate reductase; NiR, nitrite reductase; GS, glutamine synthetase; GOGAT, glutamate synthetase; GDH, glutamate dehydrogenase; * *p* < 0.05; ** *p* < 0.01.

**Table S4.** Effect of the arbuscular mycorrhizal fungus (AMF) *Rhizophagus intraradices* on photosynthetic pigment contents in *Catalpa bungei* seedlings under different nitrogen (N) concentrations.

| NH_4_NO_3_ (mM) | AMF status | Chorophyll a  (mg/g FW) | Chorophyll b  (mg/g FW) | Chorophyll a+b  (mg/g FW) | Chorophyll a/b | Carotenoids (mg/g FW) |
| --- | --- | --- | --- | --- | --- | --- |
| 0.25 | NAM | 0.66±0.10c | 0.19±0.03c | 0.86±0.13c | 3.40±0.02a | 0.15±0.02c |
|  | AM | 1.53±0.08ab | 0.46±0.03b | 1.99±0.11b | 3.31±0.02a | 0.33±0.01b |
| 10.00 | NAM | 1.54±0.18ab | 0.46±0.06b | 2.00±0.23b | 3.40±0.08a | 0.36±0.03ab |
|  | AM | 1.98±0.08a | 0.70±0.03a | 2.68±0.12a | 2.86±0.02b | 0.45±0.02a |
| 45.00 | NAM | 1.13±0.20bc | 0.37±0.06b | 1.50±0.26bc | 3.01±0.10b | 0.17±0.03c |
|  | AM | 1.19±0.27b | 0.40±0.09b | 1.60±0.36b | 3.00±0.06b | 0.21±0.06c |
| Significance | AM | ** | ** | ** | ** | ** |
|  | N | ** | ** | ** | ** | ** |
|  | AM×N | ns | ns | ns | ** | ns |

NAM, non-AMF-inoculated; AM, AMF-inoculated; Different lowercase letters within each column indicate significant differences (*p* < 0.05) among treatments. Values are means ± SE (*n* = 6). Two-way ANOVA output: ns, not significant; * *p* < 0.05; ** *p* < 0.01.

**Table S5.** Effect of the arbuscular mycorrhizal fungus (AMF) *Rhizophagus intraradices* on chlorophyll fluorescence parameters in leaves of *Catalpa bungei* seedlings under different nitrogen (N) concentrations.

| NH_4_NO_3_ (mM) | AMF status | Fv/Fm | Fv/Fo | ФPSII | rETR | NPQ | qP |
| --- | --- | --- | --- | --- | --- | --- | --- |
| 0.25 | NAM | 0.8051±0.0045d | 4.17±0.11d | 0.0802±0.0063d | 35.08±2.77d | 1.96±0.08ab | 0.1585±0.0130c |
|  | AM | 0.8134±0.0035c | 4.39±0.10c | 0.0836±0.0034cd | 36.59±1.51cd | 2.10±0.08a | 0.1659±0.0073bc |
| 10 | NAM | 0.8253±0.0018ab | 4.74±0.06ab | 0.1127±0.0059ab | 49.29±2.56ab | 1.24±0.09c | 0.1926±0.0111b |
|  | AM | 0.8300±0.0012a | 4.89±0.04a | 0.1263±0.0023a | 55.21±1.00a | 1.96±0.07ab | 0.2450±0.0068a |
| 45 | NAM | 0.8198±0.0023bc | 4.56±0.07bc | 0.0978±0.0078bc | 42.80±3.42bc | 1.97±0.10ab | 0.1881±0.0118bc |
|  | AM | 0.8249±0.0018ab | 4.72±0.06ab | 0.1169±0.0072a | 51.12±3.15a | 1.80±0.12b | 0.2320±0.0127a |
| Significance | AM | ** | ** | * | * | ** | ** |
|  | N | ** | ** | ** | ** | ** | ** |
|  | AM×N | ns | ns | ns | ns | ** | ns |

NAM, non-AMF-inoculated; AM, AMF-inoculated; Fv/Fm, maximum photochemical efficiency of PSII; Fv/Fo, potential photochemical efficiency of PSII; ФPSII, actual photochemical efficiency of PSII; rETR, relative electron transport rate through PSII; NPQ, non-photochemical quenching; qP, photochemical quenching; Different lowercase letters within each column indicate significant differences (*p* < 0.05) among treatments. Values are means ± SE (*n* = 18). Two-way ANOVA output: ns, not significant; * *p* < 0.05; ** *p* < 0.01.

**Table S6.** Pearson correlation coefficients between root hormones and root morphological parameters.

| Variable | Total root length | Root surface area | Root projected area | Root volume | Length per unit volume | Average diameter | Root tips | Forks | LF | SAF | Root activity |
| --- | --- | --- | --- | --- | --- | --- | --- | --- | --- | --- | --- |
| IAA | 0.153 | 0.206 | 0.206 | 0.252 | 0.153 | 0.174 | 0.047 | 0.052 | 0.076 | 0.098 | 0.122 |
| CTK | 0.083 | 0.113 | 0.113 | 0.143 | 0.083 | 0.147 | -0.205 | -0.136 | 0.000 | 0.067 | 0.158 |
| GA_3_ | 0.601** | 0.648** | 0.648** | 0.657** | 0.601** | 0.093 | 0.410* | 0.439** | 0.484** | 0.575** | 0.516** |
| ABA | -0.242 | -0.190 | -0.190 | -0.113 | -0.242 | 0.284 | -0.435** | -0.369* | -0.304 | -0.261 | -0.143 |
| IAA/ABA | 0.476** | 0.489** | 0.489** | 0.465** | 0.476** | -0.068 | 0.549** | 0.485** | 0.436** | 0.424** | 0.316 |
| CTK/ABA | 0.450** | 0.417* | 0.417* | 0.352* | 0.450** | -0.177 | 0.317 | 0.317 | 0.418* | 0.455** | 0.407* |
| GA_3_/ABA | 0.766** | 0.781** | 0.781** | 0.742** | 0.766** | -0.072 | 0.675** | 0.670** | 0.682** | 0.752** | 0.616** |

IAA, indole-3-acetic acid; CTK, cytokinin; GA_3_, gibberellins; ABA, abscisic acid; LF, length of fine roots (0 < d ≤ 0.5 mm); SAF, surface area of fine roots (0 < d ≤ 0.5 mm); * *p* < 0.05; ** *p* < 0.01.
